# Molecularly Engineered Hydrogel Electrolyte Embedded with Multifunctional Oxygen‐Rich Macrocyclic Units for Uniform Zinc Deposition

**DOI:** 10.1002/advs.202507377

**Published:** 2025-05-29

**Authors:** Miao Sun, Guochen Ji, Meizhi Li, Junping Zheng

**Affiliations:** ^1^ Tianjin Key Laboratory of Composite and Functional Materials School of Materials Science and Engineering Tianjin University Tianjin 300350 P. R. China

**Keywords:** aqueous zinc ion battery, hydrogel electrolyte, interfacial stability, molecular chain design, multifunctional monomer

## Abstract

Hydrogel electrolyte has emerged as an effective strategy for stabilizing zinc anode. Despite certain advancements in network design, solely relying on simple combinations of traditional polymer chains or single‐function monomers is far from satisfactory in overcoming multiple challenges faced by zinc‐ion battery. Herein, a novel multifunctional monomer, benzo‐15‐crown‐5‐acrylamide (BCAm), is designed and introduced into hydrogel network (PBCM‐HE), aiming to regulate solvation sheath structure with supramolecular macrocyclic units. Specifically, rigid benzene rings in BCAm units can stabilize conformation of crown ether and bestow PBCM‐HE excellent mechanical properties with tensile‐strength of 105 kPa and compressive‐strength of 0.6 MPa. Critically, the locally electron‐rich ether bonds in supramolecular macrocycle can optimize solvation structure of hydrated zinc ions and promote ion transport, stabilizing interface interaction between electrolyte and Zn anode. Given this, PBCM‐HE possesses outstanding ionic conductivity (61.7 mS cm^−1^) and remarkable transference number (0.86). Besides, the Zn||PBCM‐HE||MnO_2_ full cells show excellent discharge specific capacity of 290.9 mAh g^−1^ at 0.1 C with Zn uniform deposition. This work innovatively develops a novel hydrogel electrolyte network with multifunctional monomer through one‐step polymerization, providing new insights and possibilities for monomer design and selection in hydrogel electrolytes, further paving the way for exploring high‐performance electrolytes in zinc‐based devices.

## Graphical abstract

A multifunctional monomer, benzo‐15‐crown‐5‐acrylamide (BCAm) is designed, and the P(BCAm‐*co*‐AM) hydrogel electrolyte (PBCM‐HE) is prepared. This design not only enhances mechanical properties by rigid‐flexible hybrid structure, but also adjusts solvation sheath structure for stable electrolyte‐Zn anode interface. PBCM‐HE possesses excellent ionic conductivity (61.7 mS cm^−1^) and transference number (0.86), along with long‐term cycle lifespan.

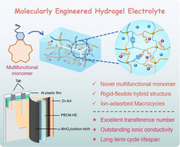

## 1 Introduction

Aqueous zinc ion battery (AZIB) has become a rising star in energy storage technology due to its high theoretical capacity (820 mAh g^−1^ and 5855 mAh cm^−3^), low redox potential of Zn anode (−0.76 V vs standard hydrogen electrode), inherent nontoxicity and high safety.^[^ ^1^, ^2^, ^3^ ^]^ However, the interfacial reaction of zinc metal is complex and unstable, which will inevitably lead to the hydrogen evolution reaction (HER),^[^ ^4^, ^5^ ^]^ thus intensifying the “tip effect” and accelerating the dendrite growth with uneven deposition of zinc ions.^[^ ^6^ ^]^ Given that the above issues are partially or wholly attributable to the interaction between the large amount of active water in electrolyte and zinc metal anode, so effective strategies must be taken to regulate electrolyte to minimize the reactivity of water.

In order to suppress zinc dendrites and side reactions, essentially speaking, it is necessary to start from regulating the structure or composition of electrolyte.^[^ ^7^, ^8^, ^9^ ^]^ One is to ensure as little free active water as possible, and the other is to optimize the solvation sheath structure of hydrated zinc ions as much as possible.^[^ ^10^ ^]^ In terms of these, the quasi‐solid hydrogel electrolyte can effectively inhibit the growth of zinc dendrites to some extent due to limited free active water.^[^ ^11^, ^12^ ^]^ Particularly, with the rapid development of wearable electronic devices under diverse application scenarios, hydrogel electrolytes can fundamentally avoid the risk of leakage compared to liquid ones.^[^ ^13^, ^14^, ^15^, ^16^ ^]^ On the other hand, regarding the strategy of regulating the solvation sheath structure, the majority of current efforts focused on introducing small molecular additives into hydrogel networks.^[^ ^17^, ^18^ ^]^ However, over‐reliance on small molecular additives may encounter risks of decomposition or concentration decay, which in turn limits the long‐term stability of battery.^[^ ^19^ ^]^ Based on the aforementioned theoretical analysis, rational structural design of network in hydrogel electrolytes instead of adding extra small molecular additives has undoubtedly become the pivotal strategy for constructing stable dendrite‐free AZIB.

As for the structural design of hydrogel network, many efforts have been made. For example, Liu et al.^[^ ^20^ ^]^ reported an optimized Zn(ClO_4_)_2_‐polyacrylamide/chitosan hydrogel electrolyte. Such a dual‐network structure endowed the hydrogel electrolyte with good anti‐fatigue performance and long lifespan of 1500 h. Ji et al.^[^ ^21^ ^]^ designed an asymmetric dual network hydrogel electrolyte with covalently cross‐linked polyacrylamide and physically crosslinked agar, realizing good tensile strength of 78.9 kPa and high compression strength of 118.0 kPa. What's more, Zhang et al.^[^ ^22^ ^]^ constructed a cross‐linked P(AM‐*co*‐SBMA) hydrogel electrolyte via free radical copolymerization of acrylamide monomer and zwitterionic SBMA monomer, and then realizing ultrahigh transfer number of 0.84 and a tensile modulus of 24.46 kPa. Throughout the previous researches, the designs of hydrogel network predominantly relied on the straightforward combination of several conventional polymer chains to construct double/semi‐interpenetrating networks, it can be conducive to enhancing mechanical properties, yet along with complicated multistep preparation methods. Alternatively, introducing the unconventional monomers with certain specific functional groups, represented by zwitterionic monomers, can be beneficial for a certain performance (such as low‐temperature performance or fast ionic migration). Unfortunately, this often comes at the expense of mechanical properties, as the salt‐like characteristics result in low flexibility and brittle hydrogels.^[^ ^22^, ^23^ ^]^ Therefore, it is imperative to engineer a novel monomer capable of integrating multiple functionalities and to fabricate hydrogel electrolyte networks via one‐step polymerization, so as to overcome the limitation of conventional monomers offering only singular functional contribution, thereby constructing high‐performance hydrogel electrolytes.

Herein, a novel hydrogel electrolyte embedded with locally highly electron‐rich supramolecular units was designed and constructed. By copolymerizing the multifunctionalized novel polymeric monomer of benzene‐15‐crown‐5‐acrylamide (BCAm) monomer with acrylamide (AM) monomer, the P(BCAm‐*co*‐AM) hydrogel electrolyte (PBCM‐HE) with single‐step polymerization was successfully prepared. The so‐called “multifunctionality” of BCAm monomer refers to possessing three distinct characteristics simultaneously: flexible and locally highly electron‐rich macrocyclic cavity, rigid benzene ring structure and radical‐polymerizable double bond. Notably, the benzo‐crown ether units incorporate both highly rotatable ether bonds and rigid benzene rings, such a rigid‐flexible hybrid structure not only stabilized the conformation of cyclic crown ether, ensuring the consistency of cavity size, but also endowed the hydrogel electrolyte with excellent mechanical properties. More significantly, the electron‐rich cyclic ether side chains within PBCM‐HE network can exhibit adsorptive effect on zinc ions, resulting in coupling between ions and polymer segments. Highly aggregated electronegative oxygen atoms in cyclic crown ethers can compete with H_2_O molecules outside the solvation sheath of hydrated zinc ions for coordination sites, optimizing the hydration structure by preferentially interacting with zinc ions, thus reducing the reactivity of active H_2_O. Furthermore, combined with the unique quasi‐solid feature of hydrogels, stable and fast zinc ion transport can be realized. In view of the innovative design of hydrogel molecular chain based on multifunctional novel monomers, a high‐performance hydrogel electrolyte without any additives was constructed, safeguarding the interface between electrolyte and anode, and realizing long cycling lifespan.

## 2 Results and Discussion

**Figure 1:**
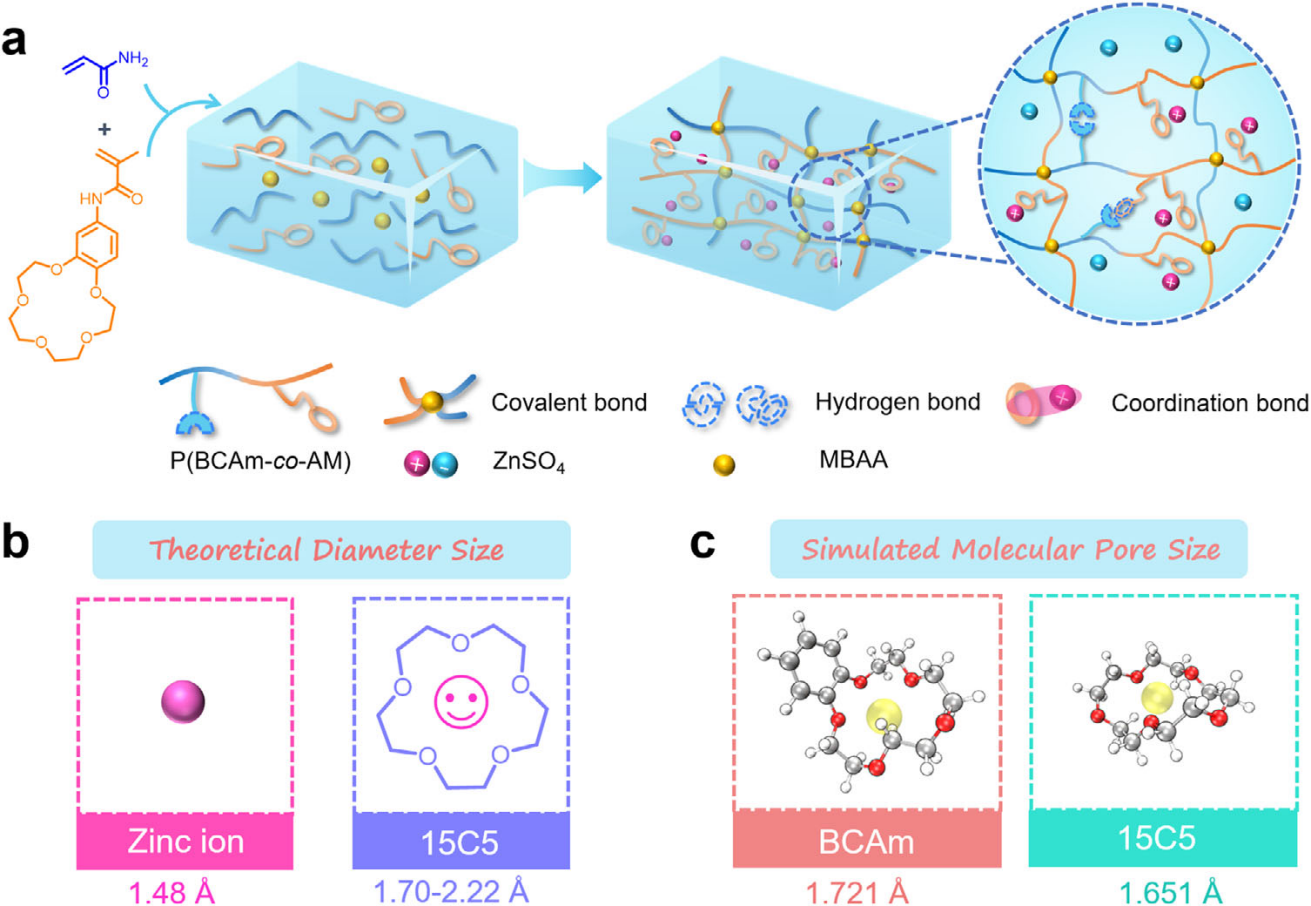
a) Schematic illustration of the synthesis of PBCM‐HE. b) Theoretical diameter size of zinc ion and 15C5 molecule. c) Simulated molecular pore size of BCAm unit and 15C5.

The preparation process of P(BCAm‐*co*‐AM) hydrogel electrolyte (PBCM‐HE) is shown in **Figure** **1**a. The crosslinked network of P(BCAm‐*co*‐AM) hydrogel (PBCM‐HG) containing supramolecular structural units was obtained by the polymerization of acrylamide (AM) monomers and benzene‐15‐crown‐5‐acrylamide (BCAm) monomers initiated by ultraviolet light with crosslinkers. Subsequently, PBCM‐HG was immersed in 2.0 m ZnSO_4_ aqueous solution (with 0.2 m MnSO_4_ as additives) for ion exchange, and finally the single‐network P(BCAm‐*co*‐AM) hydrogel electrolyte (PBCM‐HE) was prepared. In addition to covalent bonds, there are many other interactions in PBCM‐HE, including hydrogen bonds and coordination bonds. As displayed in Figure 1b, the theoretical diameter size of 15‐crown‐5‐ether (15C5) is well‐suited to match the size of zinc ion.^[^ ^24^ ^]^ However, in fact, the inherent low rotational energy barrier of ether bond endows polyether chains with significant structural flexibility,^[^ ^25^ ^]^ which may induce conformational changes and lead to the instability of macrocyclic cavity size, thereby affecting the coordination behavior of zinc ions. Notably, as shown in Figure 1c, the BCAm units have better dimensional stability with the simulated molecular pore size of 1.721 Å, which is closer to the theoretical pore size. In contrast, the pore size of 15C5 molecule is smaller than the theoretical value due to the lack of benzene ring. This phenomenon reflects the key role of introducing benzo‐crown ether unit instead of bare 15C5 unit. Profiting from the unique structure of BCAm unit, which contains both highly rotatable ether bond and rigid benzene ring, it can not only stabilize the molecular conformation of crown ether ring, but also endow electrolyte with remarkable mechanical properties. Furthermore, zinc ions can show unique transport mechanism of “adsorption‐coordination‐dissociation” in the quasi‐solid environment of PBCM‐HE, ensuring the rapid transport. More crucially, the crown ether units can effectively weaken the solvation of hydrated zinc ions and optimize the hydration structure, thus enhancing the interface stability between electrolyte and Zn anode. The ionic conductivity of PBC_x_M_y_‐HE with different monomer ratios was tested to determine the optimal composition. The electrochemical impedance spectroscopy (EIS) spectra are shown in Figure S1 (Supporting Information). The results show that the increase of benzo‐crown ether monomer can effectively reduce the charge transfer impedance. Compared with Warburg impedance slope, it can be seen that PBC_3_M_100_‐HE and PBC_5_M_100_‐HE exhibit comparable ion diffusion performance, while the charge transfer impedance of PBC_3_M_100_‐HE is slightly smaller. This may be attributed to the fact that when the monomer ratio approaches to 5:100, the steric effect of benzo‐macrocyclic units begins to appear.^[^ ^26^ ^]^ Once at this concentration, the benzo‐macrocyclic units likely induce strong local adsorption effects, causing overly tight coupling between ions and the cavities of ether oxygen, which in turn hinders the free diffusion of ions, thus limiting the further improvement of ionic conductivity to some extent. Consequently, after comprehensive consideration, the monomer ratio represented by PBC_3_M_100_‐HE is deemed the most suitable choice to achieve a balance between favorable ion diffusion properties and efficient charge transfer.

**Figure 2:**
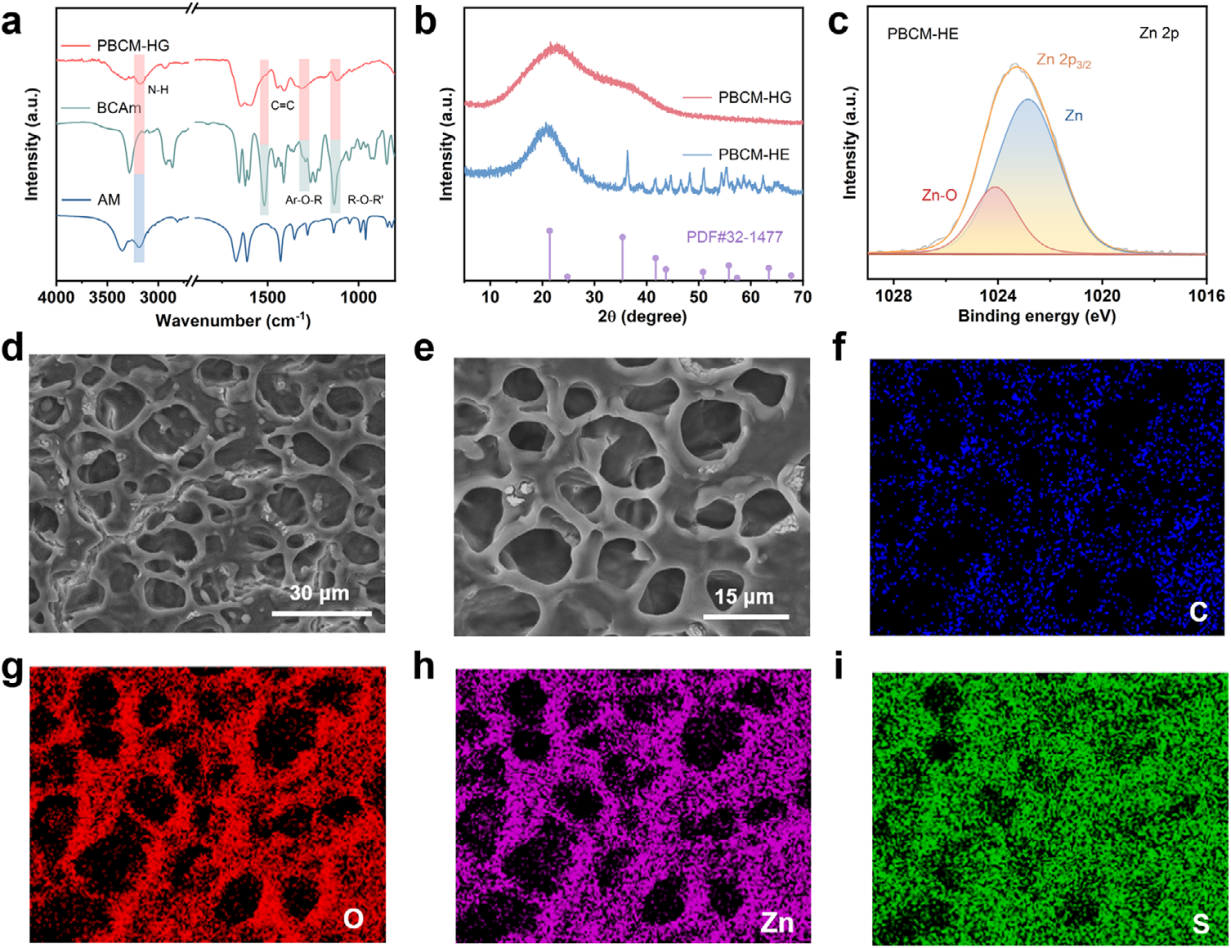
a) FT‐IR spectra of monomers and PBCM‐HG. b) XRD patterns of PBCM‐HG and freeze‐dried PBCM‐HE. c) XPS spectra of Zn 2p in PBCM‐HE. d,e) SEM images and elemental mapping of f) C, g) O, h) Zn, i) S.

The successful synthesis of BCAm monomer was confirmed by ^1^H NMR.^[^ ^27^ ^]^ As shown in Figure S2 (Supporting Information), the chemical shifts at 7.03–7.35 ppm correspond to the protons of the aromatic ring (H_a_‐H_c_), while the chemical shifts at 3.59–4.05 ppm are attributed to the protons in the crown ether ring (H_d_‐H_g_). In addition, the chemical shift at 9.62 ppm corresponds to the proton of secondary amide (H_h_), and the protons of C═C double bond (H_i_ and H_j_) are located at 5.47 and 5.77 ppm, respectively.^[^ ^28^ ^]^ As for the PBCM‐HG, the chemical structure was confirmed by FT‐IR.^[^ ^29^ ^]^ As shown in **Figure** **2**a, the characteristic peaks at 1517, 1280, and 1130 cm^−1^ are attributed to the C═C skeletal stretching vibration of phenyl ring, the C─O asymmetric stretching vibration of aromatic ether bond and the C─O asymmetric stretching vibration of R‐O‐R' in BCAm monomer, respectively. Additionally, the N─H stretching vibration corresponding to the amide group can be observed at 3187 cm^−1^. The typical characteristic peaks of BCAm monomer and AM monomer can be observed simultaneously in the FT‐IR spectrum of PBCM‐HG, indicating the successful polymerization of hydrogel. After soaking in ZnSO_4_ solution, PBCM‐HE hydrogel electrolyte was obtained. The XRD pattern in Figure 2b shows that PBCM‐HE exhibits a broad peak at ≈22°, which is the characteristic signature of amorphous materials.^[^ ^30^ ^]^ This result indicates that PBCM‐HE is primarily composed of amorphous regions, which can be attributed to the random arrangement of structural units. The amorphous structure can provide more migration channels for ions, which is beneficial to improve the ionic conductivity of electrolyte.^[^ ^31^ ^]^ Besides, corresponding diffraction peaks of ZnSO_4_ can be observed in freeze‐dried PBCM‐HE electrolyte, indicating the successful introduction of ZnSO_4_ during the electrolyte preparation. The electrostatic interaction mechanism between crown ether units and zinc ions in PBCM‐HE hydrogel electrolyte was preliminarily verified via XPS test. Prior to testing, the hydrogel electrolyte was fully soaked in deionized water, aiming to completely eliminate any possible interference of free zinc sulfate. As shown in Figure S3 (Supporting Information), the XPS spectra show the significant Zn‐O characteristic peak at 530.8 eV, which directly proves the stable ion‐dipole interaction between zinc ions and electron‐rich oxygen atoms in polymer chains,^[^ ^32^ ^]^ which is conducive to enhancing the ionic conductivity of the electrolyte. Furthermore, the high‐resolution spectra of Zn 2p shown in Figure 2c reconfirm the existence of Zn‐O characteristic peak, which can be consistent with previous reports,^[^ ^23^ ^]^ providing robust evidence for the stable transport of Zn ions within the hydrogel electrolyte. The surface morphologies of freeze‐dried PBCM‐HG hydrogel (Figure S4, Supporting Information) and PBCM‐HE (Figure 2d,e) with various scales were characterized by SEM. It can be found that PBCM‐HE presents the hierarchical pore structure, which is crucial for promoting the efficient migration of Zn^2+^ ions.^[^ ^33^ ^]^ Figure 2f–i visually show the distribution of C, O, Zn and S elements through the EDS mapping images of the selected region. The Zn elements are uniformly distributed in the whole hydrogel framework. Well‐markedly, the distribution of Zn elements is highly consistent with that of O elements, whereas the S elements from ZnSO_4_ exhibit an irregular and random distribution. This once again illustrates the interaction between zinc ions and hydrogel network, which is exactly consistent with the result of XPS.

**Figure 3:**
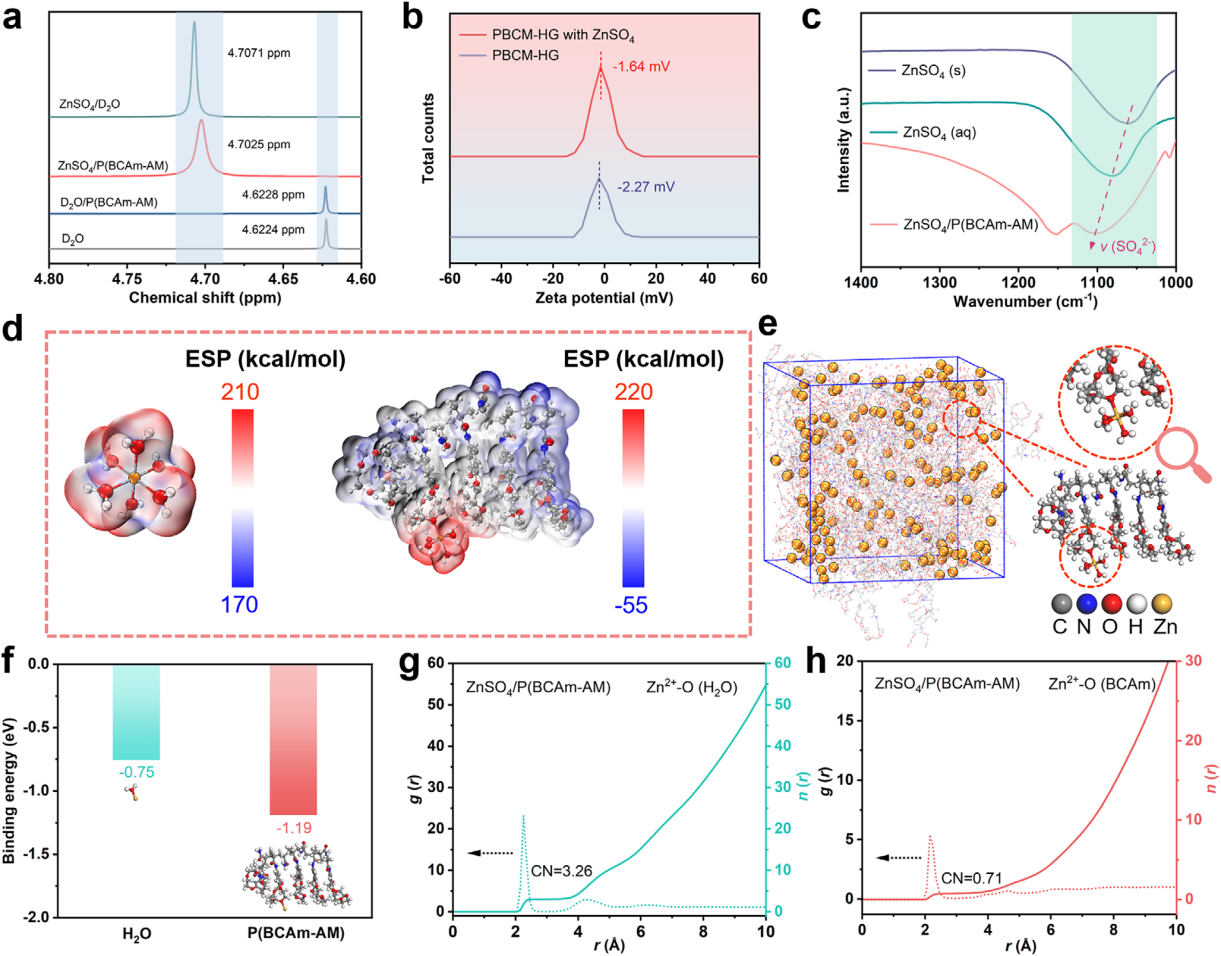
a) ^2^H NMR spectra of H_2_O from pure D_2_O, D_2_O /P(BCAm‐AM), ZnSO_4_ and ZnSO_4_/P(BCAm‐AM). b) Zeta potential of PBCM‐HG hydrogel before and after soaking in ZnSO_4_ aqueous solution. c) FT‐IR spectra of the peak shift of *v*(SO_4_^2−^) in ZnSO_4_ (s), ZnSO_4_ (aq) and ZnSO_4_/P(BCAm‐AM). d) ESP mapping of the original Zn^2+^‐6H_2_O (left) and P(BCAm‐AM)‐Zn^2+^‐5H_2_O. e) 3D snapshot of ZnSO_4_/P(BCAm‐AM) system obtained from MD simulations and local enlarged snapshot of solvation structure of zinc ion. f) Binding energy for zinc ions with H_2_O and P(BCAm‐AM) under DFT calculation. RDFs for g) Zn^2+^‐O (H_2_O) and (h) Zn^2+^‐O (BCAm unit) via MD simulations.

In order to further make clear the basic mechanism of solvation structure, a series of experimental characterizations were conducted on the electrolyte. Firstly, the NMR tests were performed on different electrolytes using D_2_O as the solvent. To ensure the feasibility of tests, uncrosslinked linear P(BCAm‐AM) was used to represent PBCM‐HE, so as to explore the effect of macrocyclic crown ether structure on solvated H_2_O molecules in PBCM‐HE. As shown in **Figure** **3**a, the ^2^H peak of pure D_2_O is located at 4.6224 ppm. After the addition of ZnSO_4_, a notable shift in the peak location to 4.7071 ppm can be observed. This is attributed to the solvation‐induced coordination between zinc ions and water molecules, which subsequently leads to the decreased surrounding electronic density.^[^ ^34^ ^]^ While after P(BCAm‐AM) was added, the chemical peak slightly moved back to 4.7025 ppm, which implies that the electron‐rich crown ether units in the polymer participated in the coordination environment of Zn^2+^, releasing part of limited H_2_O molecules to ensure the reactivity is not too high. In addition, for rigorous comparison, the P(BCAm‐AM) was directly dissolved with D_2_O without ZnSO_4_. It can be found that the ^2^H peak reverts to 4.6228 ppm, further indicating that the coordination between PBCM‐HE and zinc ions caused the variation of chemical shift. Due to the abundance of active sites provided by the electron‐rich cavities within the network, PBCM‐HG exhibits a negative Zeta potential of −2.27 mV, as shown in Figure 3b. Upon the addition of ZnSO_4_, the Zeta potential shifts to −1.64 mV, indicating the adsorption effect of the hydrogel toward zinc ions, which is conducive to the desolvation of zinc ions during the deposition process.^[^ ^35^, ^36^ ^]^ Furthermore, the FT‐IR spectra confirm that PBCM‐HE can weaken the solvation of Zn^2+^ with H_2_O. As shown in Figure 3c, the vibration stretching of SO_4_^2−^ (*v*(SO_4_^2−^)) in ZnSO_4_ powder is located at ≈1060 cm^−1^, while it undergoes a significant blue shift to 1081 cm^−1^ after being dissolved in H_2_O. This is due to the formation of the primary solvation shell under the aqueous environment, which can weaken the interaction between zinc ions and sulfate ions.^[^ ^37^ ^]^ After adding P(BCAm‐AM), *v*(SO_4_^2−^) shifts to higher wave numbers, indicating a weakened electrostatic coupling between Zn^2+^ and SO_4_^2−^, which reduces the confinement effect surrounding SO_4_^2−^. This result serves as compelling evidence in favor of the viewpoint that PBCM‐HE can effectively modulate the solvation shell of zinc ions, thereby influencing the interaction dynamics in electrochemical process. Moreover, the adsorption test of zinc ions by PBCM‐HE was briefly explored as shown in Figure S5 (Supporting Information). The concentration of zinc ions in zinc sulfate solutions before and after adding hydrogel was measured by inductively coupled plasma emission spectrometry (ICP‐OES) with the testing condition consistently maintained at 25 °C and a pH of 6.0. It can be found that the concentration of zinc ions decreased from the initial 59.07–44.63 mg L^−1^ after the addition of PBCM‐HG hydrogel.^[^ ^38^ ^]^ The decreasing trend of the concentration of zinc ions indicates strong adsorption efficiency for zinc ions of the PBCM‐HG hydrogel. Additionally, the electrostatic potential (ESP) distribution of Zn^2+^‐6H_2_O and P(BCAm‐AM)‐Zn^2+^‐5H_2_O solvation structures were provided for comparison. As shown in Figure 3d, the P(BCAm‐AM)‐Zn^2+^‐5H_2_O structure exhibits lower ESP accompanied by gradient charge distribution. This result indicates that the migration of zinc ions within the PBCM‐HE hydrogel electrolyte exhibits notable kinetic advantages.^[^ ^39^ ^]^ Molecular dynamics (MD) simulation was employed to analyze the solvation structure of ZnSO_4_/P(BCAm‐AM) system. The statistical result (Figure 3e) show that the electron‐rich macrocycles within the P(BCAm‐AM) molecular chains have replaced the original H_2_O molecule, demonstrating that the introduction of P(BCAm‐AM) chains has significantly regulated the solvation structure.^[^ ^40^ ^]^ Notably, based on the ion adsorption effect induced by electron‐rich cavities, zinc ions do have specific coordination with oxygen atoms in crown ether structure. In addition, due to the conjugated structure of rigid benzene ring, BCAm units exhibit parallel stacking arrangement within the P(BCAm‐AM) chains, which plays a positive role in enhancing the strength and stability of PBCM‐HE hydrogel electrolyte. The interactions between H_2_O, BCAm, units and zinc ions were evaluated by quantum chemical calculation. As shown in Figure 3f, the binding energy of zinc ions with P(BCAm‐AM) is higher than that with H_2_O, revealing that BCAm units tend to coordinate with Zn^2+^ preferentially to adjust the solvation environment. To further analyze the solvation structure, the corresponding radial distribution function (RDF) in ZnSO_4_/P(BCAm‐AM) electrolyte were obtained. As shown in Figure 3g,h, Zn^2+^‐O peaks from H_2_O and BCAm unit appear at ≈2 Å away from Zn^2+^, revealing that BCAm unit entered the original solvation sheath of hydrated zinc ions and participated in the regulation of solvation structure.^[^ ^41^ ^]^ All the above test results and molecular dynamics simulations collectively confirm that the electron‐rich macrocyclic structure incorporated into the side chain of hydrogel network can effectively reduce the solvation interaction between zinc ions and H_2_O under the ion‐dipole interaction between ions and polymer segments.

**Figure 4:**
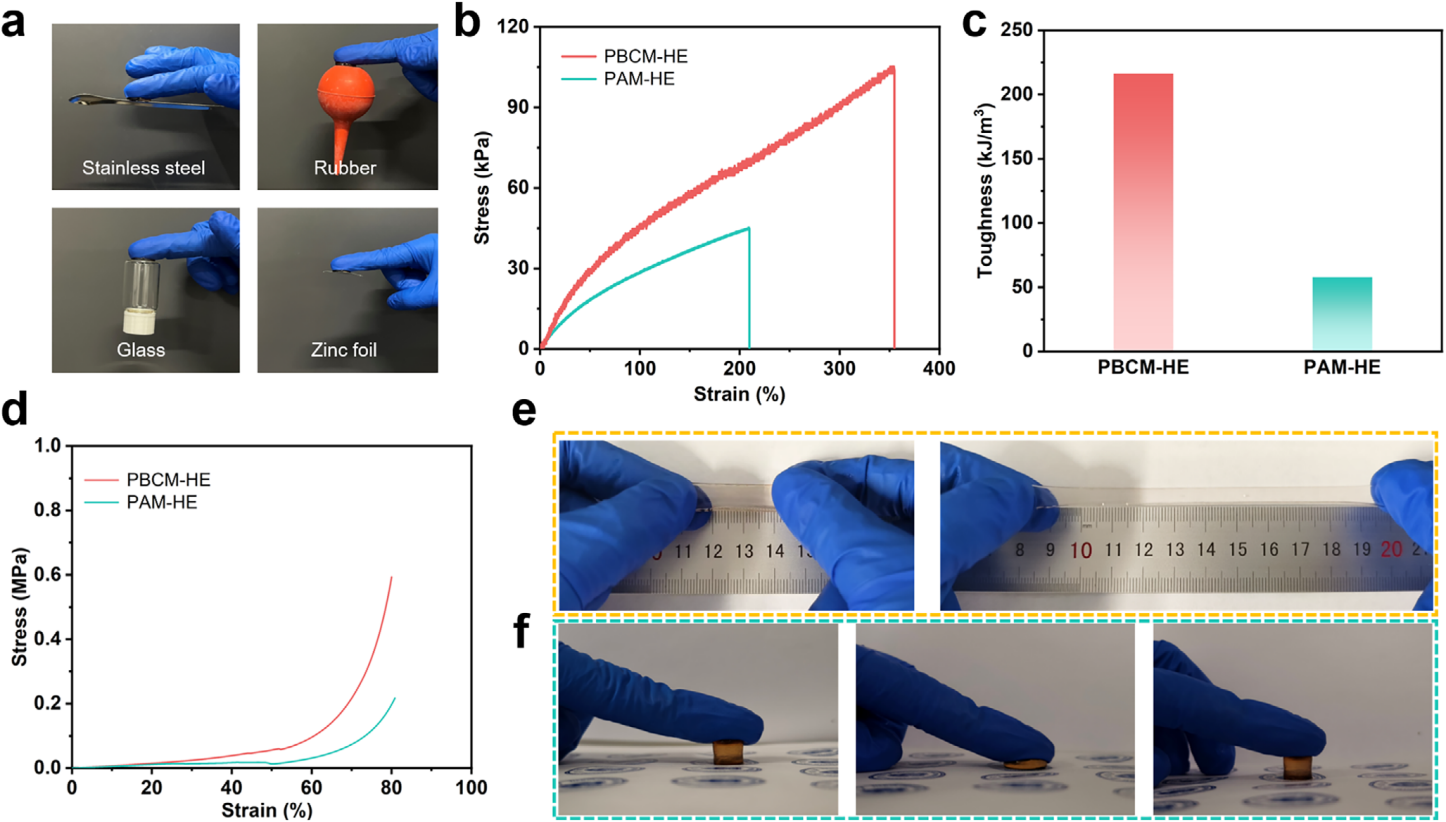
a) Adhesive properties of PBCM‐HE on various materials. b) The tensile stress‐strain curves and c) Corresponding toughness of two hydrogel electrolytes. d) The compressive stress–strain curves of PBCM‐HE and PAM‐HE. Digital images of e) tensile and f) compressive properties of PBCM‐HE.

The excellent interfacial adhesion of PBCM‐HE on various surfaces (stainless steel, rubber, glass, and zinc foil) is demonstrated in **Figure** **4**a, showing its broad compatibility and potential applications. The mechanical properties of hydrogel electrolyte were evaluated. As shown in Figure 4b, the fracture stress of PBCM‐HE can be up to 105 kPa with a fracture strain of 353%, which is 2.4 times of the fracture strength of PAM‐HE, significantly outperforming the hydrogel electrolyte without rigid‐flexible supramolecular macrocyclic structure. Specifically, the highly flexible ether bonds in the cyclic crown ether cavities can facilitate to disperse the stress and improve the toughness (Figure 4c), while the rigid structure of benzene rings can provide stable frameworks, which significantly enhanced the mechanical properties of PBCM‐HE hydrogel electrolyte. On the other hand, as shown in Figure 4d, PBCM‐HE exhibits remarkable compression resistance, capable of enduring extreme deformation (up to 80% compressive strain) without cracking. The corresponding compression stress is up to 0.6 MPa. Besides, intuitive digital images of tensile and compressive properties are also shown in Figure 4e,f, which further visually proves the exceptional mechanical properties of PBCM‐HE. In order to further verify the flexibility of PBCM‐HE hydrogel electrolyte, some extreme tests were carried out in Figure S6 (Supporting Information). The results show that it has excellent flexibility without damage and cracking even under bending and twisting state. In addition, we also compare the mechanical properties of PBCM‐HE with other reported works. It can be seen that, compared with other single‐network structural hydrogel electrolytes in Table S1 (Supporting Information), PBCM‐HE has outstanding fracture strength and compressive strength, which are even higher than those of some double‐network structural hydrogel electrolytes.

**Figure 5:**
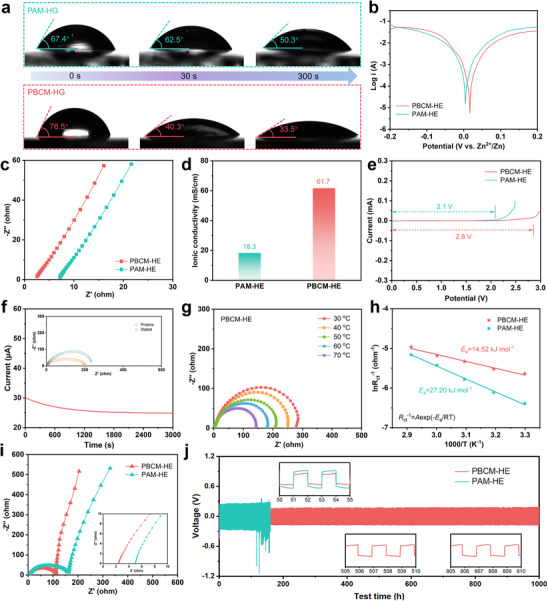
a) Contact angles of ZnSO_4_ electrolyte on PAM hydrogel and PBCM hydrogel. b) Tafel curves of PBCM‐HE and PAM‐HE electrolytes. c) Nyquist plots and d) calculated ionic conductivities of two hydrogel electrolytes. e) LSV curves. f) Zn^2+^ transference number characterization of PBCM‐HE. g) Nyquist plots of Zn||PBCM‐HE||Zn cell at various temperatures. h) Arrhenius curves and the corresponding calculated desolvation activation energies of two hydrogel electrolytes. i) EIS spectra. j) Comparison of voltage‐time curves of Zn||Zn symmetric cells in two electrolytes under the current density of 1.0 mA cm^−2^.

Dynamic contact angle measurements were employed to evaluate the surface affinity of zinc sulfate electrolyte with various hydrogels in **Figure** **5**a. Intriguingly, it can be distinctly observed that the electrolyte droplet rapidly transformed from the initial spherical shape into a flat liquid film on the surface of PBCM‐HG within the same time compared to PAM hydrogel. This phenomenon is attributed to the unique supramolecular macrocyclic structural units in PBCM‐HG which exhibits strong affinity for zinc ions, thereby promoting intimate contact between the ZnSO_4_ electrolyte and hydrogel surface. Additionally, the incorporation of hydrophilic cavities in PBCM‐HG further enhances the hydrophilicity of surface, enabling the electrolyte droplet to propagate more readily and fully cover the entire surface. This underscores the high affinity of PBCM‐HG toward zinc sulfate solution, not only providing a specific binding environment for zinc ions but also modulating surface wetting behavior through its distinctive spatial configuration. Prior to a series of electrochemical tests, the thickness of PBCM‐HE was compared with that of commercial glass fiber (Figure S7, Supporting Information). The highly transparent PBCM‐HE can be observed from the inset in Figure S7a (Supporting Information). Furthermore, there is almost no difference in the measured thickness between the two, with a slight difference of only 0.09 mm. The electrochemical corrosion behavior of zinc anodes in PBCM‐HE and pure PAM‐HE hydrogel electrolytes was evaluated by Tafel polarization test. The more positive corrosion potential (*E*_corr_) signifies the smaller tendency for hydrogen evolution reaction (HER).^[^ ^42^, ^43^ ^]^ Additionally, the lower corrosion current (*I*_corr_) indicates the lower rate of HER. As shown in Figure 5b, PBCM‐HE, containing cyclic polyether, obviously exhibits a more positive *E*_corr_ and a lower *I*_corr_ of 5.53 µA compared to PAM‐HE, demonstrating the superior electrochemical stability and enhanced corrosion resistance of zinc anodes within the PBCM‐HE. Then, the conductivity of two hydrogel electrolytes was measured and calculated. The EIS spectra in Figure 5c show that PBCM‐HE electrolyte exhibits smaller interfacial impedance and higher ion diffusion efficiency. According to $\sigma =\frac{L}{R_{b}\times S}\;$ formula,^[^ ^44^ ^]^ the ionic conductivity of PBCM‐HE was calculated to be 61.7 mS cm^−1^, which is more than three times that of PAM‐HE (Figure 5d), revealing the positive role of benzo‐crown ether units in promoting ion transport. The electrochemical stability window of hydrogel electrolyte was evaluated via the linear sweep voltammetry (LSV) method. As shown in Figure 5e, PBCM‐HE exhibits significantly enhanced electrochemical stability, with a wider electrochemical window of 2.8 V, indicating its potential for application in a wide voltage range. The Zn^2+^ transfer number of PBCM‐HE was further determined by measuring Zn||Zn symmetrical cell with a constant polarization potential. The corresponding *i*‐*t* curve and EIS spectra before and after polarization are shown in Figure 5f. The Zn^2+^ transfer number was calculated according to the formula^[^ ^45^ ^]^: $t_{Zn^{2+}}=\frac{I_{s}\left(\Delta V-I_{0}R_{0}\right)}{I_{0}\left(\Delta V-I_{s}R_{s}\right)}\;$, where Δ*V* represents the polarization potential of 10 mV, *I*_0_ and *I*_s_ are the pristine and stated current, *R*_0_ and *R*_s_ are the charge transfer resistance (*R*_ct_) measured by EIS. According to this formula, the $t_{Zn^{2+}}$ value of PBCM‐HE is as high as 0.86, indicating the remarkable advantage of PBCM‐HE in Zn^2+^ migration. This may be attributed to the continuous complexation‐decomplexation process between crown ether units and the target zinc ions, which can accelerate the migration rate of Zn ions in the electrolyte, thus realizing the efficient diffusion of Zn^2+^.

In order to further explore the desolvation energy barrier mechanism of solvation structure regulated by cyclic crown ether at the electrode/electrolyte interface, EIS tests were conducted to further investigate the Arrhenius plots and activation energies (*E*_a_) of symmetric cells employing various hydrogel electrolytes under different temperatures, as shown in Figure 5g,h. Specifically, the *E*_a_ can be calculated by the classical Arrhenius equation: $\frac{1}{R_{\mathrm{ct}}}=A_{0}\;exp\left(-\frac{E_{a}}{RT}\right)$. Among them, the parameters involved in the equation include charge transfer resistance (*R*_ct_, Ω), pre‐exponential factor (*A*_0_), activation energy (*E_a_*, kJ mol^−1^), molar gas constant (R, J mol^−1^ K^−1^) and Kelvin temperature (*T*, K). Moreover, Figure S8 (Supporting Information) presents the EIS curves of symmetric cells with PAM‐HE hydrogel electrolyte at different temperatures. The results show that the *R_ct_* with PBCM‐HE is significantly lower than that of PAM‐HE, strongly proving the superior interfacial compatibility of PBCM‐HE hydrogel electrolyte. Notably, the activation energy (14.52 kJ mol^−1^) of PBCM‐HE is significantly lower than that of PAM‐HE (27.20 kJ mol^−1^), revealing the unique advantages of PBCM‐HE in promoting the rapid desolvation kinetics of solvated zinc ions, which can be conducive to the more uniform deposition of zinc ions.^[^ ^46^, ^47^, ^48^ ^]^ The EIS spectra of the full cells with two electrolytes shows significant differences in Figure 5i. Specifically, PBCM‐HE exhibits a smaller semicircle diameter in the high‐frequency region corresponding to a lower *R*_ct_ of 108.5 Ω, significantly lower than that of PAM‐HE (*R*_ct_ = 164.0 Ω). Additionally, PBCM‐HE exhibits the smaller ohmic resistance (*R*_ohm_, corresponding to the intercept on the real axis), indicating the exceptional compatibility at the electrode/electrolyte interface, which can further promote the rapid transport of zinc ions between the two phases. Zn plating/stripping tests were carried out under galvanostatic charge/discharge process in Zn||Zn symmetrical cells to evaluate the impact of various electrolytes on the performance of the zinc anode. The voltage‐time curves are shown in Figure 5j under a current density of 1.0 mA cm^−2^ with a fixed charge‐discharge time of 1 h. Notably, the PAM‐HE electrolyte exhibits significant voltage fluctuation after merely 118 h of cycling, ultimately leading to battery failure. By contrast, the Zn||Zn symmetrical battery of PBCM‐HE can cycle stably for more than 1000 h with lower overpotential throughout the whole cycling. Moreover, Zn plating/stripping performance at higher current density was tested, as shown in Figure S9 (Supporting Information). It can be observed that PBCM‐HE hydrogel electrolyte can withstand higher current density, and it can still achieve stable Zn plating/stripping even at a current density of 10 mA cm^−2^. These excellent performances are attributed to the introduction of benzo‐crown ether units, which not only promotes the selective transport of zinc ions, but also significantly enhances the diffusion efficiency of zinc ions by optimizing the electrolyte structure and effectively reduces the overpotential, thereby improving the overall energy efficiency of the battery. Furthermore, the Coulombic efficiency (CE) of zinc stripping/plating was evaluated using the Zn||Cu asymmetric cell operated at the areal capacity of 1 mAh cm^−2^.^[^ ^49^ ^]^ Of note, the Zn||PBCM‐HE||Cu cell demonstrated impressive electrochemical reversibility, achieving an average CE of 98.4% over extended cycling (Figure S10, Supporting Information) with highly overlapping plating‐stripping curves.

**Figure 6:**
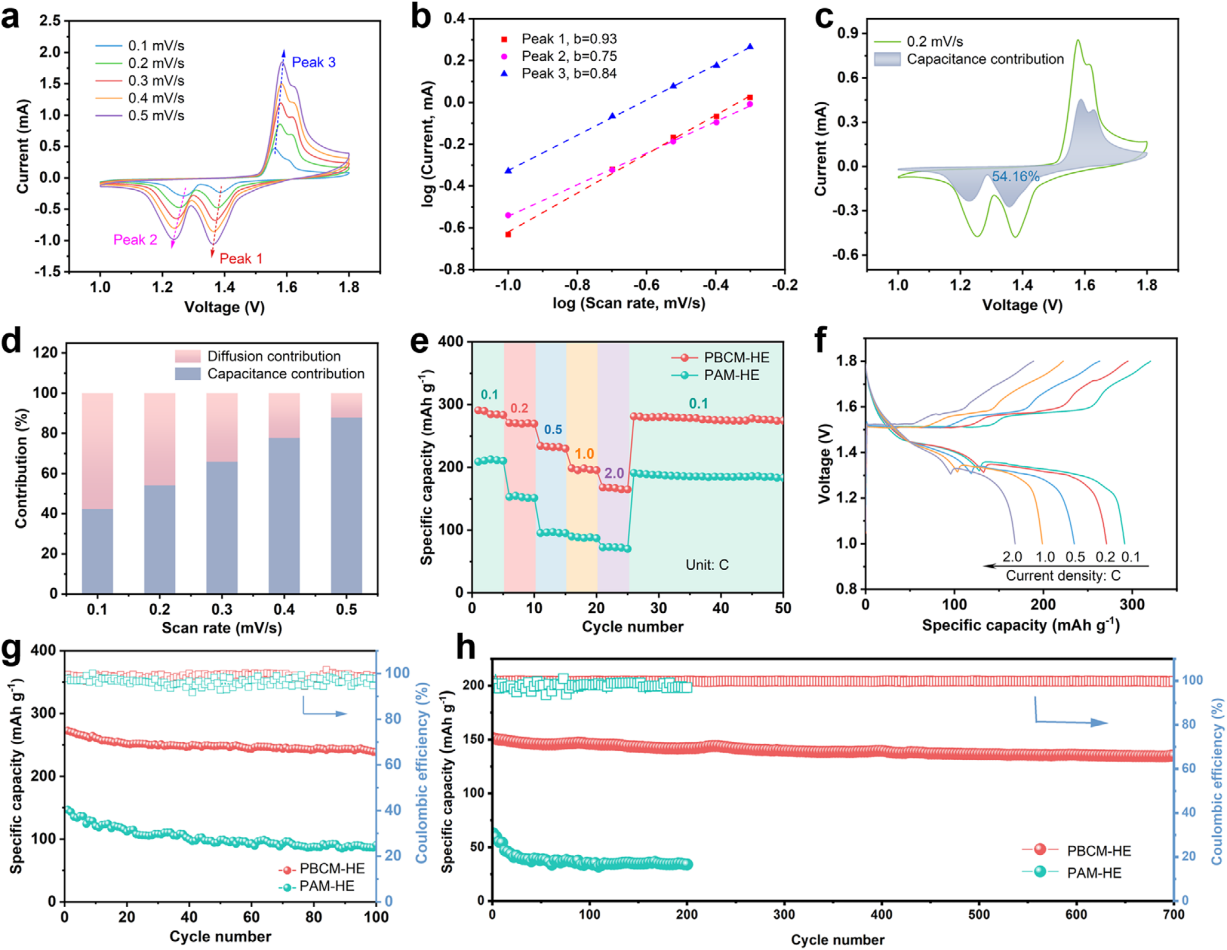
a) CV curves of PBCM‐HE at various scanning rates. b) b values of corresponding redox peaks. c) Capacitance contribution (blue part) and diffusion contribution (blank part) at 0.2 mV s^−1^. d) Capacitance contribution ratios at various scan rates. e) Comparison of rate performance with various hydrogel electrolytes. f) GCD curves under different C‐rate of PBCM‐HE. g) Cycling performance at 0.2 C of various hydrogel electrolytes. h) Long cycling performance at 5.0 C of PBCM‐HE and PAM‐HE.

Cyclic voltammetry (CV) measurements were conducted at different scanning rates to further analyze the intrinsic electrochemical kinetics of PBCM‐HE as shown in **Figure** **6**a. The obtained CV curves exhibit two pairs of redox peaks with slight shifts in voltage peaks as the increase of scanning rate. The correlation between peak current (*i*) and scanning rate (*v*) can be mathematically formulated as *i*  =  a*v* ^b^, where a and b are two empirical constants. Then the logarithm of both sides in the formula can be converted into: log(*i*) = log(a)  + blog(*v*), facilitating analysis and interpretation of the relationship between peak current and scanning rate. Through the linear fitting of log(*i*) and log(*v*), the b values of cathodic and anodic Peaks 1–3 are calculated in Figure 6b. As universally acknowledged, b values of 1 and 0.5 correspond to capacitive and diffusion‐controlled reactions, respectively.^[^ ^50^ ^]^ The peak values of Peak1‐3 were measured at 0.93, 0.75, and 0.84, respectively. This suggests that the charge storage mechanism during the electrochemical reaction involves both surface adsorption/desorption of ions and ion diffusion processes. The contribution of capacitive and diffusive behaviors was quantitatively evaluated by the formula *i*  = k_1_ *v*+k_2_ *v* ^1/2^, where k_1_ *v* represents capacitive capacity and k_2_ *v* ^1/2^ corresponds to diffusion‐controlled capacity. Based on the linear relationship of $\frac{i}{v^{\frac{1}{2}}}=k_{1}\;v^{1/2}+k_{2}$, the parameters of k_1_ and k_2_ can be obtained, which are crucial for studying the energy storage mechanism. As shown in Figure 6c, the capacitive contribution ratio reaches 54.16% at a scanning rate of 0.2 mV s^−1^, indicating the diffusion‐dominated process. This result emphasizes the favorable ionic conductivity and efficient chemical reaction rate in AZIB, which is conducive to the rapid ion transport and electrochemical transformation. The other capacity contribution ratios corresponding to different scanning rates are shown in Figure S11 (Supporting Information). As the scan rate increases to 0.5 mV s^−1^ in Figure 6d, the capacitive contribution ratio increased significantly to 87.90%. This result arises from the diffusion‐limited behavior at higher scan rate, while the capacitive behavior (such as surface adsorption/desorption and rapid oxidation‐reduction reaction) can complete the storage and release of charges in a shorter time, offering a more efficient kinetic response.^[^ ^51^ ^]^ This transition from diffusion dominance to capacitance dominance not only shows that the cell has excellent charge storage capacity under high‐rate condition, but also signifies its potential for realizing rapid charge‐discharge cycling and high‐rate capacity retention in practical applications. The rate performance of hydrogel electrolyte was evaluated from 0.1 C to 2.0 C (Figure 6e), and it can be found that PBCM‐HE cells show excellent specific capacity at different rates. As the current density increased, the specific capacity gradually decreased, with values of 290.9, 270.8, 234.2, 198.5, and 167.9 mAh g^−1^ at 0.1, 0.2, 0.5, 1.0, and 2.0 C, respectively. Particularly, upon the C‐rate returning to the initial 0.1 C, the specific capacity can still be maintained up to nearly 281.2 mAh g^−1^, demonstrating remarkable electrochemical reversibility. Furthermore, galvanostatic charge‐discharge (GCD) curves under different rates are depicted in Figure 6f, which conforms to the typical GCD curves of Zn‐MnO_2_ battery. Specifically, during the discharge process, the curve shows a sloping plateau at ≈1.4 V, followed by an extended flat plateau at ≈1.3 V. These two characteristic plateaus correspond to the insertion processes of H^+^ and Zn^2+^ ions,^[^ ^52^ ^]^ directly confirming the two‐step electrochemical reaction mechanism in the battery, which is highly consistent with the CV results. The electrochemical cycle stability was evaluated as shown in Figure 6g. The PBCM‐HE cell can achieve a high initial discharge capacity of 272.5 mAh g^−1^ at a rate of 0.2 C, which can still remain stable at 238.7 mAh g^−1^ after 100 cycles, significantly outperforming the unmodified PAM‐HE. Besides, the GCD curves corresponding to different cycle numbers are shown in Figure S12 (Supporting Information). In addition, the PBCM‐HE cell possesses stable cycle performance at the high rate of 5.0 C (Figure 6h). After 700 cycles, it could still maintain a specific capacity of 135.1 mAh g^−1^, representing a capacity decay of 0.015% per cycle. In contrast, the unoptimized PAM‐HE electrolyte demonstrates low capacity and rapid decay, achieving a capacity of only 34.0 mAh g^−1^ after 200 cycles. In addition, we compared the battery performance based on the PBCM‐HE hydrogel electrolyte with other reported works, as shown in Table S2 (Supporting Information). The results show that the Zn‐MnO_2_ full cell based on PBCM‐HE exhibits relatively excellent initial discharge specific capacity. After long‐term cycling tests, it can still maintain a satisfactory specific capacity, which fully demonstrates the reliability and stability of the PBCM‐HE electrolyte.

**Figure 7:**
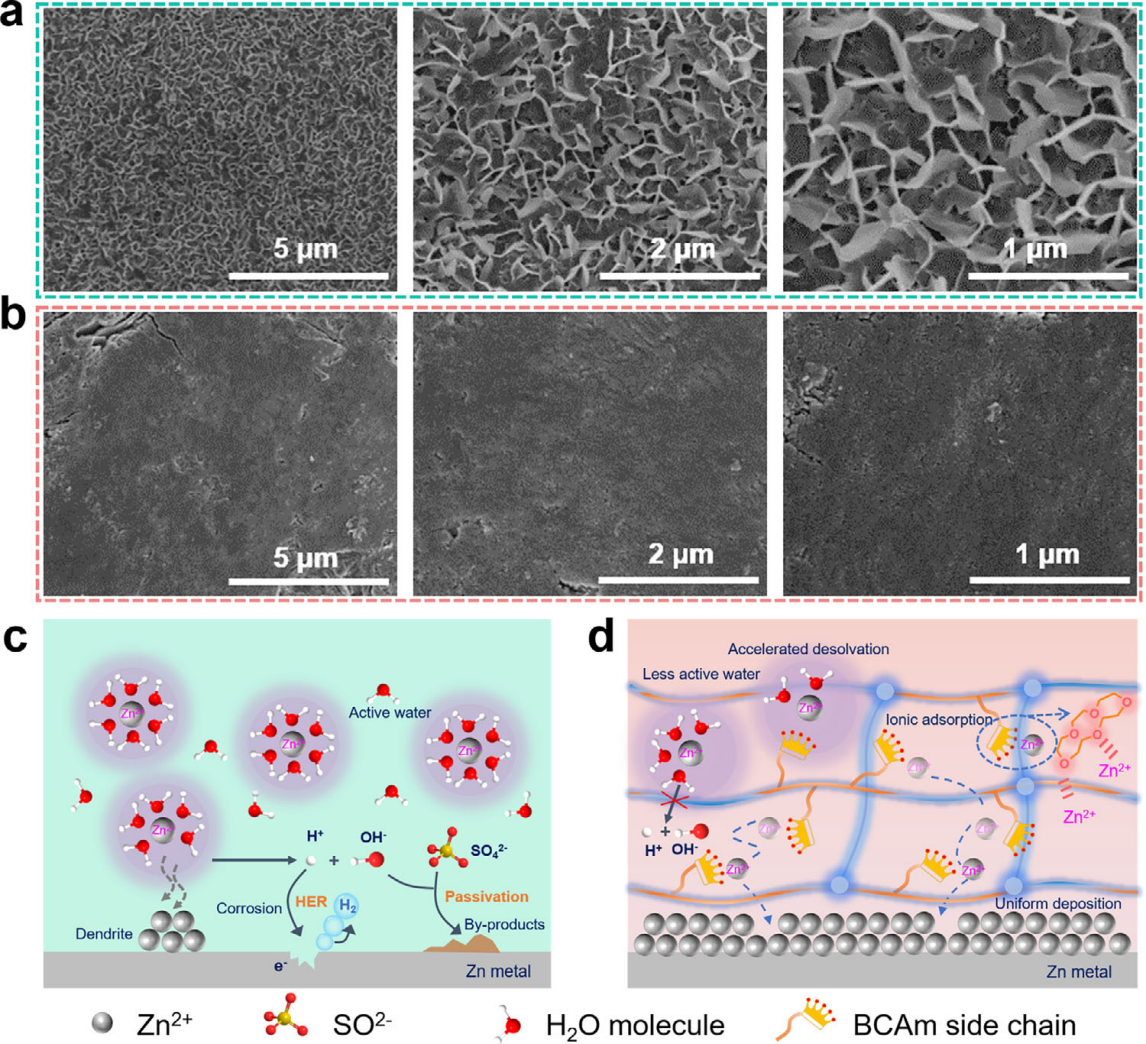
The SEM morphologies of Zn foils after cycling in a) PAM‐HE and b) PBCM‐HE. Schematic diagram of internal mechanism between zinc anode and electrolyte interface with c) pure ZnSO_4_ and d) PBCM‐HE.

In order to investigate the inhibitory effect of the hydrogel electrolyte containing cyclic crown ether structural units (PBCM‐HE) on zinc dendrite growth, the morphologies of zinc anode surfaces based on PAM‐HE and PBCM‐HE after multiple charge‐discharge cycles were studied by SEM, as shown in **Figure** **7**a,b. The results show that in traditional PAM‐HE, the zinc foil exists obvious Zn dendrite, leading to extremely uneven surface and accompanied by severe accumulation of by‐products. In stark contrast, the zinc anode in the PBCM‐HE, which incorporates cyclic crown ether structural units, displays distinctly different morphological characteristics. The surface remains highly smooth with almost no corrosion, and no observable Zn dendrite formation. This strongly proves the remarkable ability of the designed PBCM‐HE in regulating and stabilizing the chemical environment at the Zn anode interface. The positive mechanism of PBCM‐HE hydrogel electrolyte in electrochemical process is illustrated visually by Figure 7c,d. In conventional ZnSO_4_ electrolyte, zinc ions are predominantly in the form of highly solvated hydrated Zn^2+^, leading to the aggregation of a large number of active H_2_O molecules at the interface between Zn metal and electrolyte.^[^ ^53^ ^]^ Such highly active water molecules are easily decomposed into H^+^ and OH^−^, in which H^+^ will corrode the zinc metal anode accompanied by HER, while accumulated OH^−^ tends to form the passivation layer on the anode surface, significantly compromising the reversibility of cycling process.^[^ ^54^ ^]^ More critically, given that the overpotential of zinc nucleation is notably higher than that of crystal growth, Zn^2+^ will preferentially deposit at regions with higher surface energy (“tip effect”), resulting in the deterioration of zinc dendrites.^[^ ^55^, ^56^ ^]^ In stark contrast, the incorporation of cyclic crown ether units in the PBCM‐HE hydrogel electrolyte can optimize the solvation structure of zinc ions, effectively mitigating the reactivity of water molecules at the interface. Concurrently, the unique zincophilic ether oxygen groups on the polymer side chains play a pivotal role by not only accelerating the rapid transport of zinc ions but also inducing the smooth and uniform deposition on the anode surface, thereby significantly inhibiting the formation of zinc dendrites.

**Figure 8:**
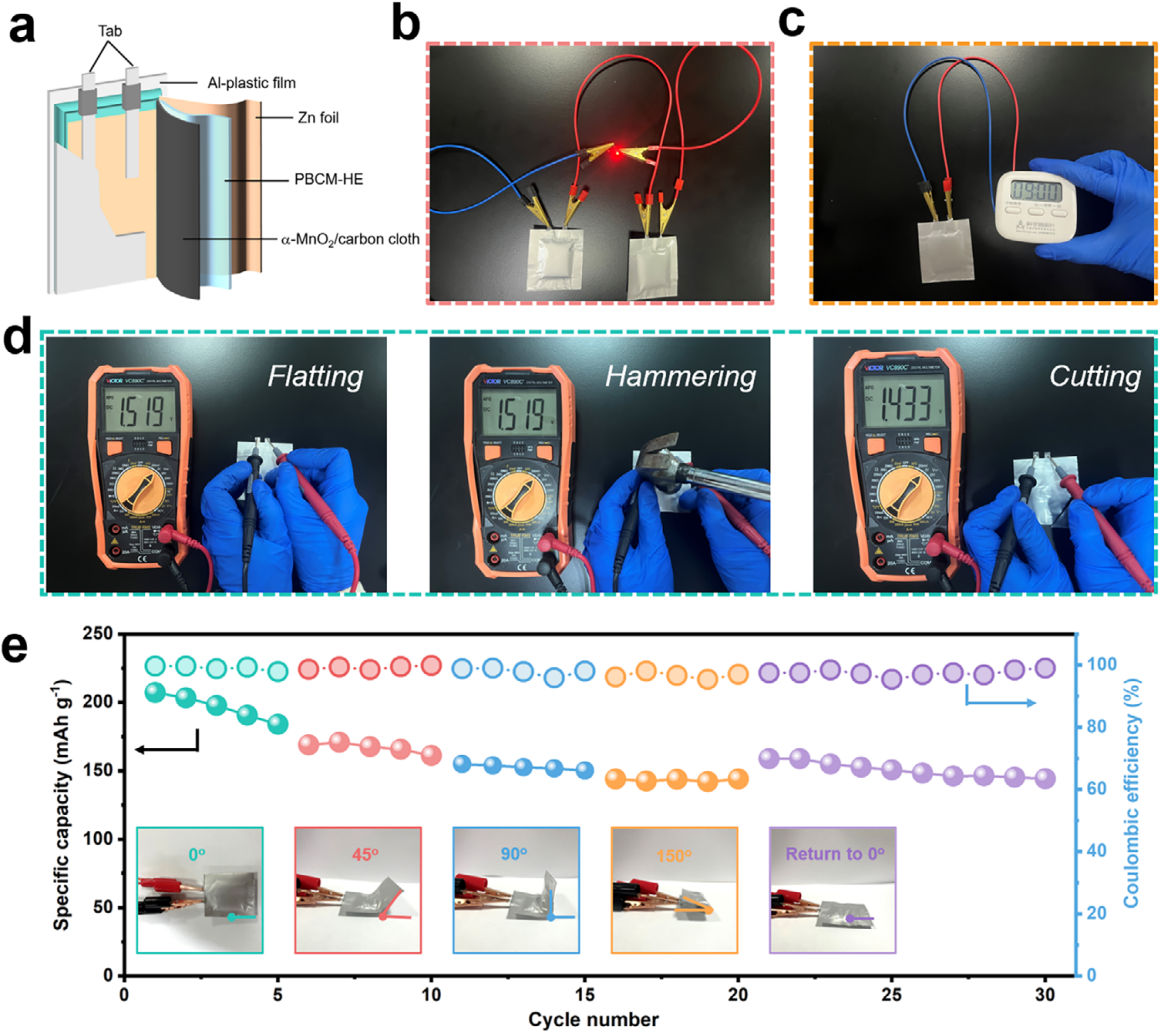
a) Schematic diagram of assembled Zn||PBCM‐HE||MnO_2_ pouch battery. Digital images of b) LED and c) electronic timer powered by flexible pouch battery. d) Digital images of open‐circuit voltages of pouch battery with PBCM‐HE under various external forces. e) Cycling performance of flexible battery under different bending angles at 0.2 C.

As shown in **Figure** **8**a, the flexible pouch battery was assembled with PBCM‐HE as electrolyte, and subsequently encapsulated with Al‐plastic film. This assembled pouch battery shows stable power output capability, successfully illuminating the LED light (Figure 8b) and efficiently powering an electronic timer to ensure its continuous and reliable operation (Figure 8c), thereby confirming its potential and feasibility in practical applications. In addition, the voltage stability of flexible battery under complex environment was evaluated by multimeter, as shown in Figure 8d. Notably, the flexible battery exhibits minimal voltage fluctuations upon bending, hammering, and even cutting. Besides, the specific capacity of the flexible battery under different bending angles was tested. As depicted in Figure 8e, the specific capacity remained relatively stable, indicating the reliability of the PBCM‐HE flexible battery in practical applications, portending promising potential in the field of portable and wearable devices.

## 3 Conclusion

In summary, a PBCM‐HE hydrogel electrolyte embedded with multifunctional supramolecular macrocyclic units was designed and prepared. Through the rational design of molecular chain, the structure of solvent sheath was modulated, effectively circumventing the decomposition risks and concentration fluctuations that may arise from traditional small molecular additives. The uniqueness of benzo‐crown ether unit lies in the combination of highly rotatable ether bonds and rigid benzene rings, which can not only stabilize the molecular conformation of crown ether ring, but also impart excellent tensile (105 kPa) and compressive (0.6 MPa) properties to the electrolyte. In this way, a stable and flexible ion transport channel network was skillfully constructed, thus promoting stable and rapid transport of zinc ions. As a result, the PBCM‐HE has excellent ionic conductivity (61.7 mS cm^−1^) and high zinc ion transfer number (0.86). It is particularly noteworthy that the locally electron‐rich ether bond environment can optimize the solvation structure of hydrated zinc ions, significantly inhibiting the side reactions induced by active water decomposition. The symmetrical cell based on PBCM‐HE can cycle stably over 1000 h without obvious zinc dendrites. Even at the high current density of 5.0 C, the specific capacity of Zn‐MnO_2_ full cell can still be maintained 135.11 mAh g^−1^ after 700 cycles, showing extremely low cycle capacity decay rate (only 0.015% per cycle). In addition, the extreme condition tests on flexible pouch cells further verified the stable voltage output of the full cells based on PBCM‐HE. Through rational molecular chain design, this study successfully constructed a novel hydrogel electrolyte network embedded with multifunctional electron‐rich macrocycles and clarified its intrinsic mechanisms through a series of characterizations and theoretical calculations. This work provides a strategic guideline for developing new reaction monomers for hydrogel electrolyte with specific functional groups and chemical structures, thereby paving the way for precise regulation over hydrogel electrolytes.

## 4 Experimental Section

A detailed Experimental Section can be found in the supporting information.

## Conflict of Interest

The authors declare no conflict of interest.

## Supporting information



Supporting Information

## Data Availability

The data that support the findings of this study are available from the corresponding author upon reasonable request.
