# Supplementary material for: Molecularly Engineered Hydrogel Electrolyte Embedded with Multifunctional Oxygen‐Rich Macrocyclic Units for Uniform Zinc Deposition
Source: Adv Sci (Weinh). 2025 May 29;12(32):e07377. doi: 10.1002/advs.202507377 (PMC12407374; doi:10.1002/advs.202507377)
Supplement: Supplementary file 1 — Supporting Information [file ADVS-12-e07377-s001.docx]

Supporting Information

Molecularly Engineered Hydrogel Electrolyte Embedded with Multifunctional Oxygen-Rich Macrocyclic Units for Uniform Zinc Deposition

Miao Sun, Guochen Ji, Meizhi Li, Junping Zheng*

Tianjin Key Laboratory of Composite and Functional Materials, School of Materials Science and Engineering, Tianjin University, Tianjin 300350, People's Republic of China.

E-mail: jpzheng@tju.edu.cn

**Experimental Section**

***Materials***

Acrylamide (AM) monomer, 4'-aminobenzo-15-crown-5, α-ketoglutaric acid, zinc sulfate heptahydrate (ZnSO_4_⋅7H_2_O, AR) and methacryloyl chloride were purchased from Aladdin Biochemical Technology Co., Ltd (Shanghai, China). These raw materials were used directly without further processing. Triethylamine (TEA), n-hexane, dichloromethane (DCM) and N, N'-methylenebisacrylamide (MBAA, AR) were all obtained from Meryer Chemical Technology Co., Ltd. (Shanghai, China). Potassium permanganate (KMnO_4_, AR), sulfuric acid (H_2_SO_4_, AR) and N-methyl-2-pyrrolidone (NMP, AR) were supplied by Jiangtian Chemical Technology Co., Ltd. (Tianjin, China). Zn foil, PVDF binder (battery grade) and Super P were purchased from Canrd New Energy Technology Co., Ltd. (Guangdong, China).

***Synthesis of*** ***Benzo-15-crown-5-acrylamide (******BCAm) Monomer***

The BCAm monomers were obtained through the amidation reaction of 4'-aminobenzo-15-crown-5, and the specific steps are as follows: 4’-aminobenzo-15-crown-5 (0.300 g, 1.059 mmol) was dissolved together with TEA (0.214 g, 2.118 mmol) in DCM (3 mL). The mixture was cooled to 0 ^o^C, and then methacryloyl chloride (0.221 g, 2.118 mmol) was added dropwise to the mixture with stirring. The mixture was maintained at 0 ^o^C for 30 min, followed by slowly raising the temperature to room temperature, and reacted for 16 h. After the reaction, the solution was precipitated with n-hexane, filtered, and dried under vacuum at 25 ^o^C for 24 h to obtain the final powdered product.

***Synthesis of P(BCAm-co-AM) Hydrogel Electrolyte (PBCM-HE)***

The BCAm monomers were dissolved in deionized water and stirred for 10 min under nitrogen atmosphere. Subsequently, AM monomers and MBAA crosslinker (1 mol%) were added and thoroughly mixed into the solution. Then, 1 wt.% of ultraviolet initiator (α-ketoglutaric acid) was added to the solution and fully stirred. The prepolymer solution was then injected into a mold composed of two parallel glass plates and a silicone rubber gasket. And the radical polymerization was carried out under ultraviolet irradiation for 15 min to obtain PBCM hydrogel (PBCM-HG). According to the different molar ratio of the two monomers, PBC_x_M_y_ hydrogels with different composition ratios were prepared. In this process, the prepolymer solution can not be completely converted into hydrogel state even after extending the ultraviolet irradiation time to 2 h when the molar ratio of BCAm exceeded 5 mol%, due to the inherent steric hindrance of the macrocyclic structure. Finally, the obtained hydrogels were immersed in 2.0 M ZnSO_4_ aqueous solution (with 0.2 M MnSO_4_ as additives) overnight for sufficient ion exchange, thus obtaining PBCM hydrogel electrolyte (PBCM-HE).

***Preparation of α-MnO_2_ Nanofibers***

α-MnO_2_ nanofibers were prepared via conventional hydrothermal method, following previous work.^[1]^ Initially, 3 mmol of MnSO_4_ and 1 mol of H_2_SO_4_ were dissolved in 30 mL of deionized water with stirring continuously. Subsequently, 20 mL of aqueous solution containing 0.1 M KMnO_4_ was added drop by drop. The mixture was heated at 120 °C for 12 h in a Teflon-lined autoclave. After cooling, the product was washed with water/ethanol and dried under vacuum at room temperature.

***Material Characterizations***

The FT-IR spectroscopy was conducted using Nicolet IS20 spectrometer (Thermo) within a wavenumber range spanning from 400 to 4000 cm^-1^. The NMR analysis was carried out with AVANCE IIIHD 800 MHz spectrometer (Bruker) with DMSO-*d*_6_ as solvent. The X-ray diffraction (XRD) measurements were conducted by D8 Advanced diffractometer equipped with Cu Kα radiation. The elemental composition and valence states of various elements were analyzed through X-ray photoelectron spectroscopy (XPS) with Thermo Scientific K-Alpha instrument. The morphological characterization of samples was conducted using the scanning electron microscope (SEM, Hitachi S4800). The Zeta potential measurements were characterized using the Malvern Zetasizer Nano ZS ZEN3600 instrument. The affinity between ZnSO_4_ electrolyte and different hydrogels was measured by a contact angle meter (Lauda Scientific LSA100). The tensile and compressive properties were evaluated by mechanical testing on a CMT4203 machine (MTS) under air atmosphere. The dimensions of the tensile test specimens were standardized at 20.0 × 10.0 × 1.0 mm^3^ with the tensile rate of 10.0 mm min^-1^. For the compressive test, cylindrical samples of 20 mm height and 10 mm diameter were compressed at a steady rate of 5.0 mm min^-1^.

***Cell Assembling Method***

The cathode was prepared by mixing the prepared α-MnO_2_ nanofibers, carbon black, and PVDF binder in a weight ratio of 7:2:1 in NMP to form uniform slurry, followed by coating it onto carbon cloth. The as-prepared composite cathode was dried at 60 ^o^C for 12 h, and then cut into wafers with a diameter of 10 mm for battery assembly. The Zn foil with a thickness of 50 μm was polished, followed by ultrasonic cleaning with ethanol, and was subsequently cut into wafers of 12 mm in diameter. The CR2032 coin cells were assembled utilizing α-MnO_2_ as cathode, hydrogels soaked in zinc sulfate solution as both the electrolyte and separator, and Zn foil as anode. For the Zn||Zn symmetrical cells, Zn foils were employed as both the working and counter electrodes, while the hydrogel immersed in ZnSO_4_ solution (with 0.2 M MnSO_4_ as additive) acted as the electrolyte.

***Electrochemical Measurements***

For electrochemical analysis, series of characterizations including cyclic voltammetry (CV), electrochemical impedance spectroscopy (EIS) and linear sweep voltammetry (LSV) were carried out on the CHI660E electrochemical workstation. Specifically, the CV test was performed under various scanning rates within a potential window of 1.0-1.8 V. EIS measurements were conducted in the frequency range from 0.01 to 100 kHz. The LSV tests were conducted with a scanning rate of 1 mV s^-1^ in a two-electrode system, featuring stainless-steel foil serving as the working electrode and Zn foil simultaneously acting as both the reference and counter electrodes. The galvanostatic charge-discharge (GCD) curves were measured on a multichannel testing system (CT-4008Tn-5V20mA, Neware), maintaining a voltage limit between 1.0-1.8 V.

***Theoretical Calculations***

In this work, molecular dynamics simulations were performed using Gromacs 2020.6 software.^[2]^ A system consisting of 50 P(BCAm-AM) polymer units containing crown ether structures (with a polymerization degree of 5), 2000 water molecules, and 150 ZnSO_4_ molecules was placed in a 10 nm × 10 nm × 10 nm box. The force field, temperature, simulation time, and time step used in the simulation were the GAFF (General Amber Force Field),^[3]^ 298.15 K, 100 ns, and 1 fs, respectively. During the total simulation time of 100 ns, the first 0-20 ns were designated as the heating phase, where the temperature increased from 0 K to 298.15 K. And the subsequent 80 ns were the constant temperature phase, during which the system temperature was maintained at 298.15 K. This phase was also used for data analysis of the system. Additionally, xtb 6.4.1 software ^[4]^ and Multiwfn software ^[5]^ were employed in this work to analyze the electrostatic potential of Zn^2+^-6H_2_O and P(BCAm-AM)-Zn^2+^-5H_2_O complexes, the molecular pore size of BCAm and 15C5, and the binding energy between P(BCAm-AM) polymer functional fragments and zinc ions. The radial distribution function (RDF), binding energy (*E*_b_), and molecular polarity index (MPI) ^[6]^ were calculated according to the following formulas:

*g*(r)=(1/*ρ*)·(*dN*(*r*)/4π*r*^2^*dr*) (1)

Where *g*(r) is the radial distribution function, *ρ* is the average number density of particles, *dN*(r) is the number of particles in a spherical shell of radius *r* and thickness *dr*, and 4π*r*^2^*dr* is the volume of the spherical shell.

*E*_b_=*E*_A+B_－*E*_A_－*E*_B_ (2)

Where *E*_A+B_, *E*_A_, and *E*_B_ were the electron energy of compound A+B, compound A, and compound B, respectively.


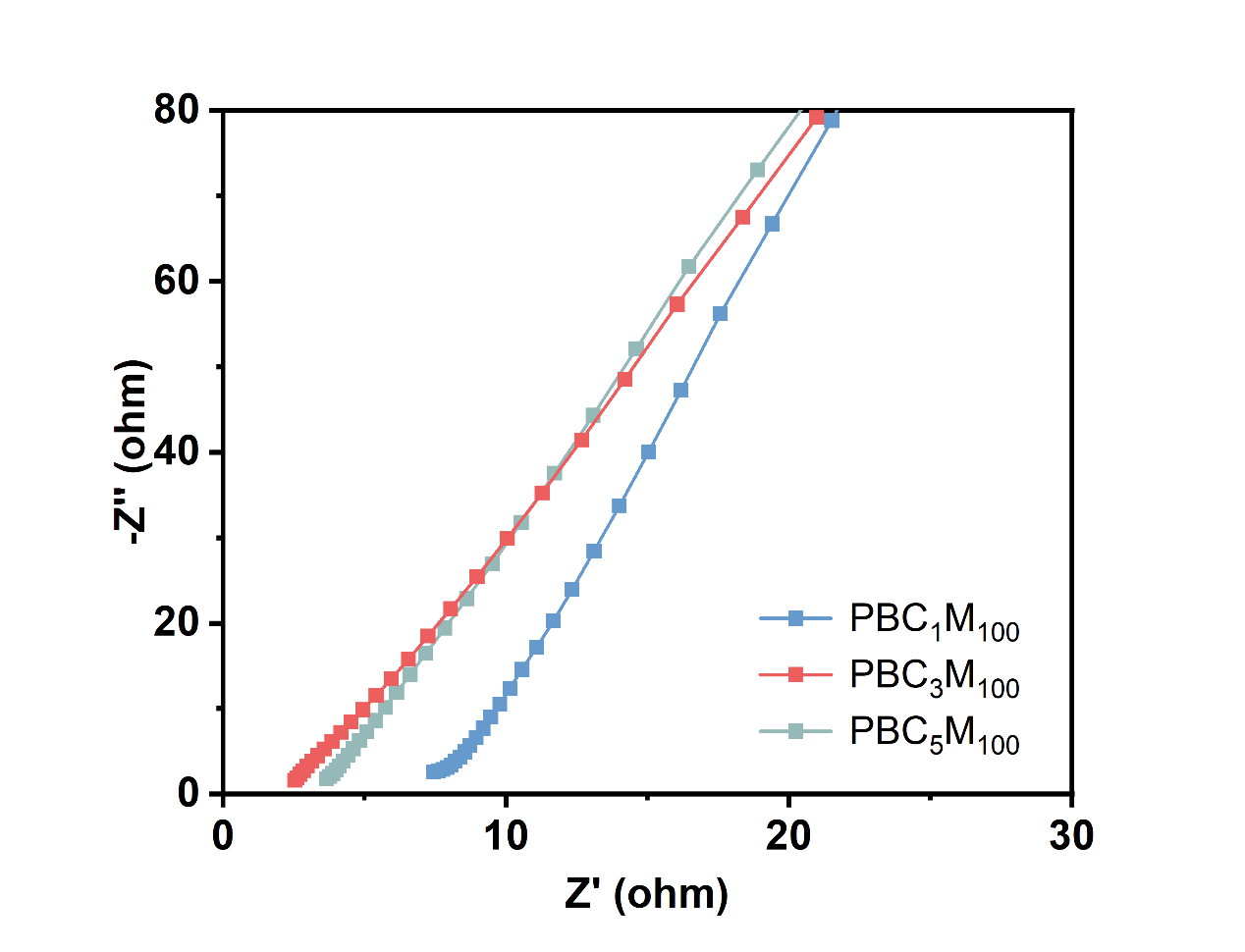


**Figure S1.** Nyquist plots of PBCxMy-HE with different monomer ratio.
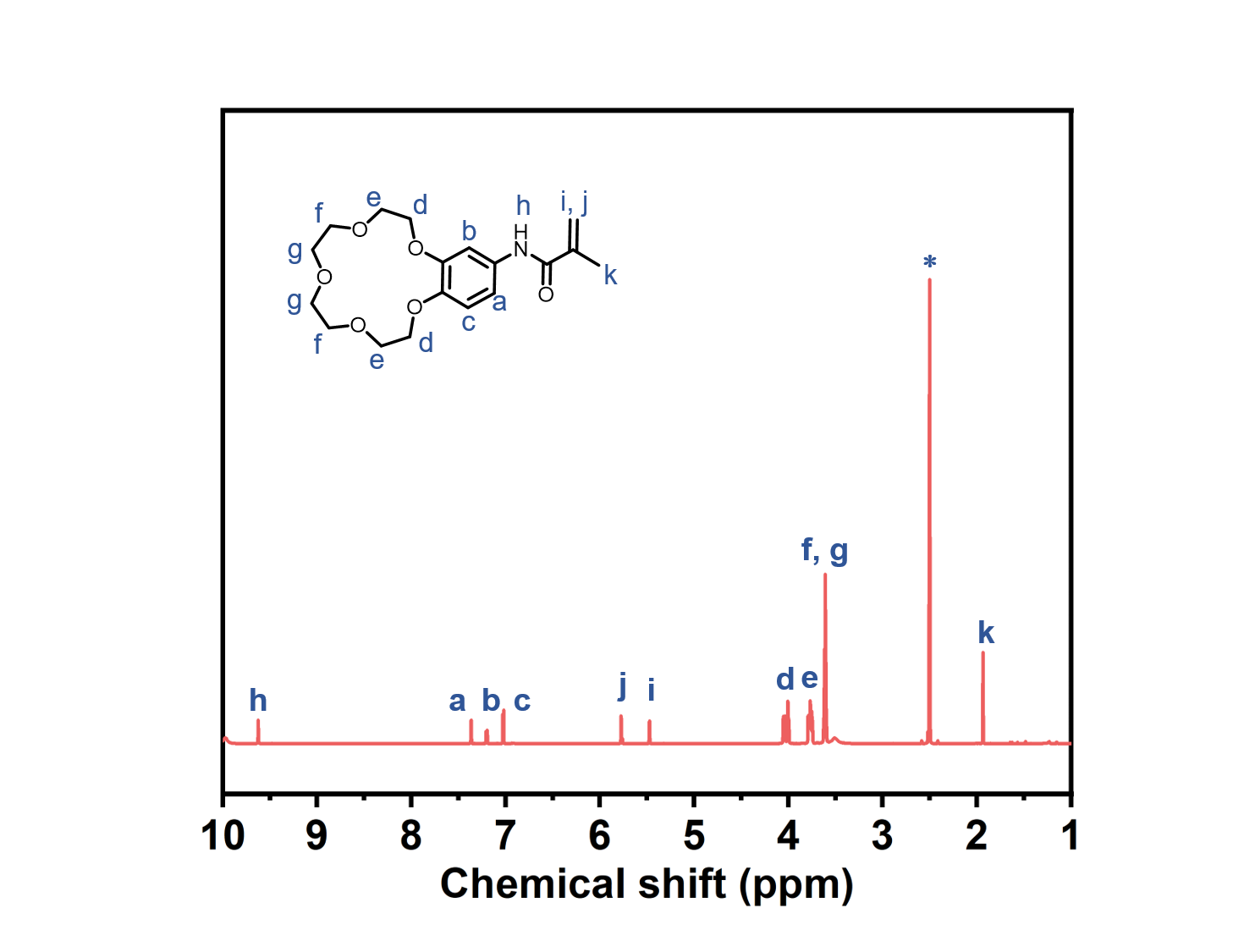


**Figure S2.** ^1^H NMR spectra of BCAm monomer.


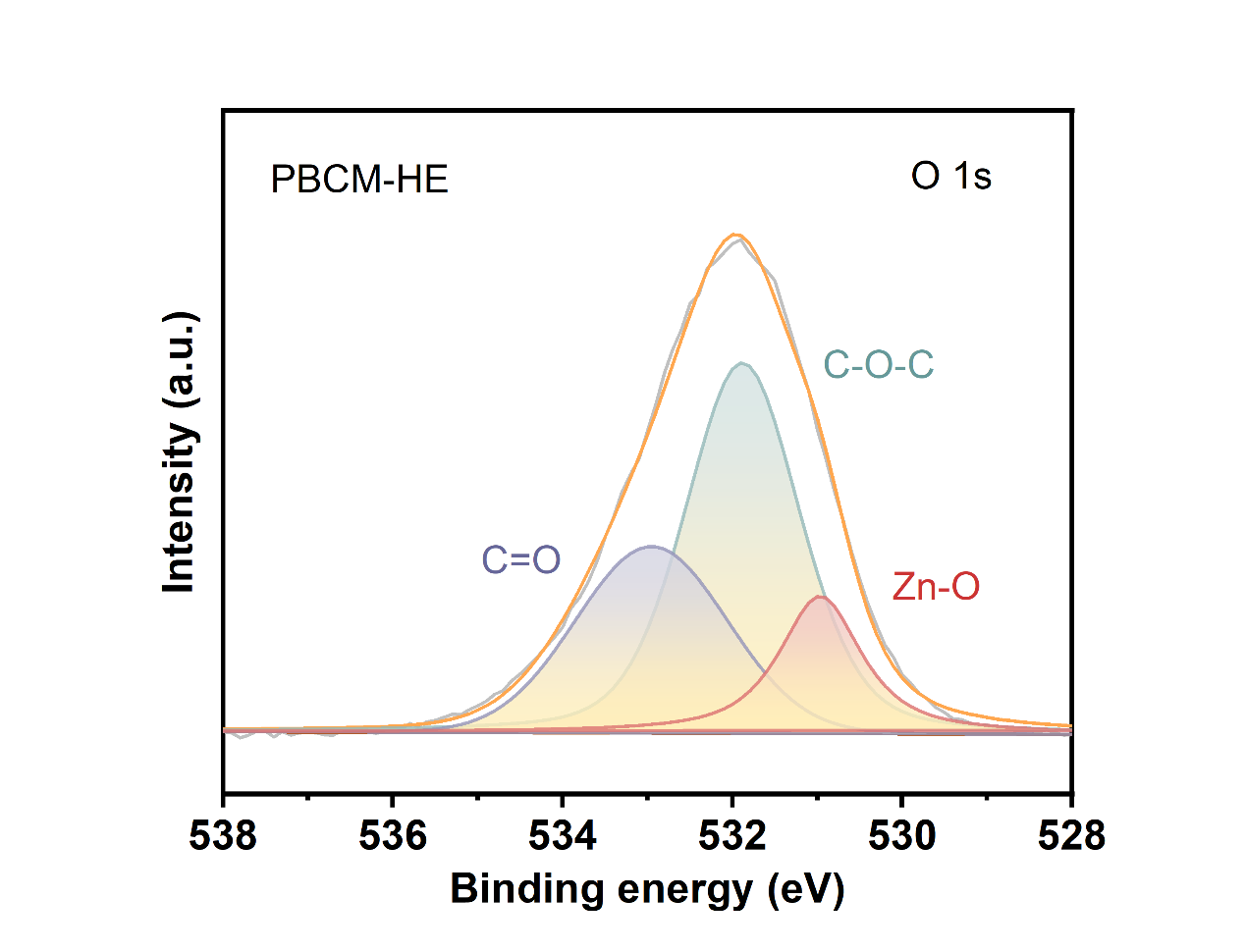


**Figure S3.** XPS spectra of O 1s in PBCM-HE.


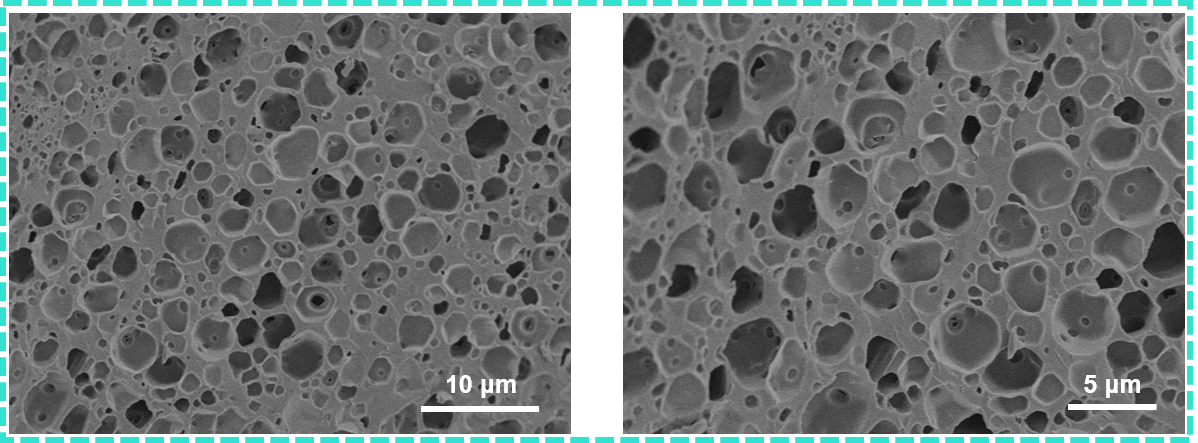


**Figure S4.** SEM images of freeze-dried PBCM-HG hydrogel.


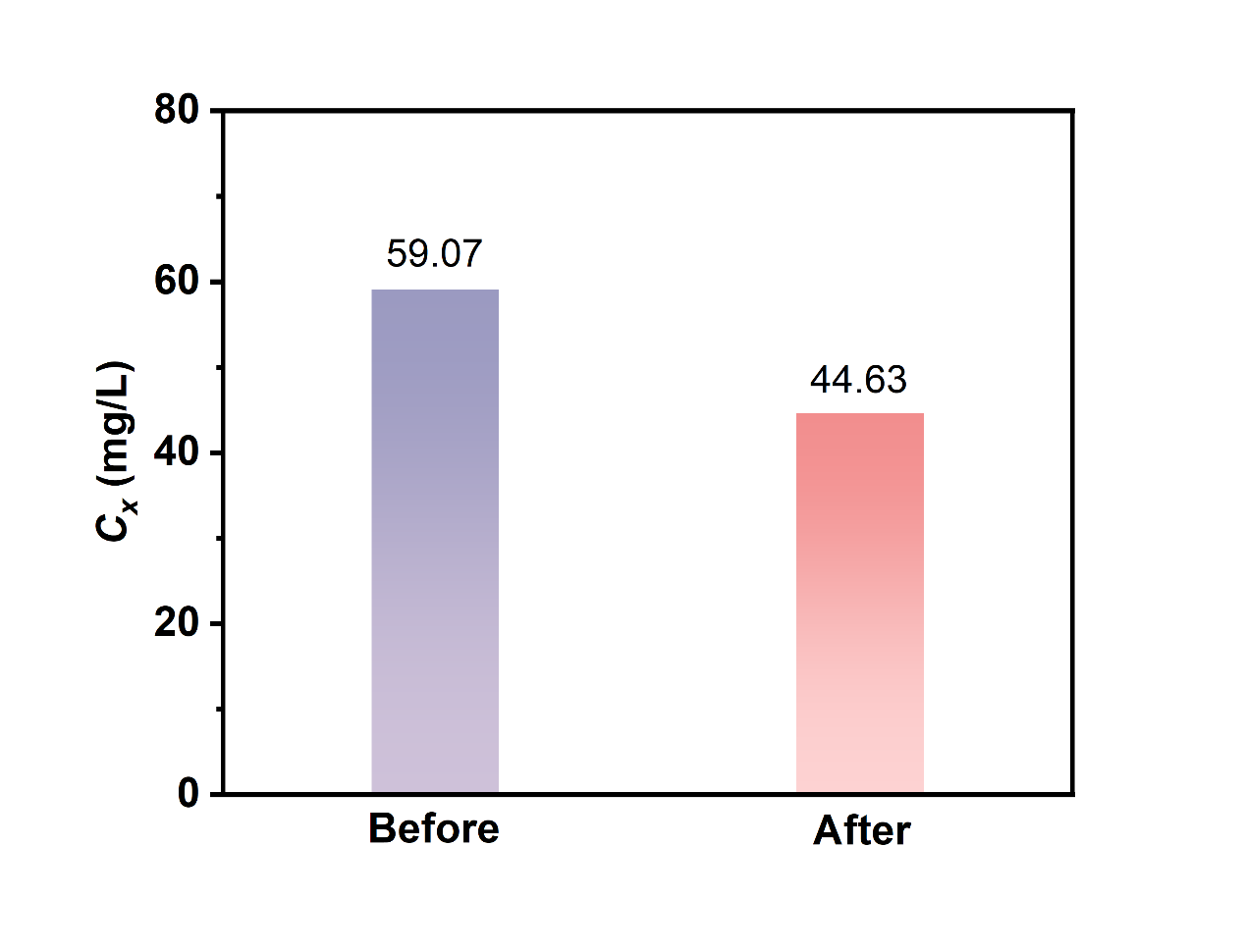


**Figure S5.** The adsorption behavior towards Zn^2+^ in PBCM-HE tested by ICP-OES.


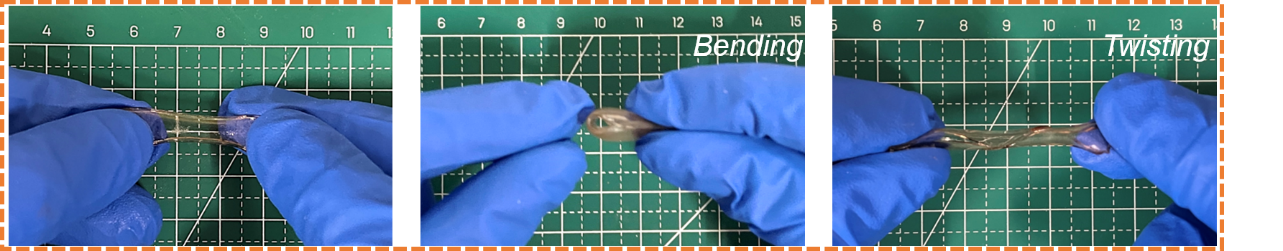


**Figure S6.** Digital photos of flexibility tests of PBCM-HE under bending and twisting.

**Table S1.** Comparison of mechanical properties between PBCM-HE work and other hydrogel electrolytes. (SN, *s*-IPN and DN stand for single network, *semi*-interpenetrating network and double network, respectively)

| **Hydrogel matrix** | **Network structure** | **Electrolyte solution** | **Fracture stress** | **Compressive properties** | **Reference** |
| --- | --- | --- | --- | --- | --- |
| PAM | SN | ZnSO_4_  LiCl | ~61 kPa | - | Adv. Funct. Mater. 2020, 30, 1907218 |
| PAM | SN | Zn(CF_3_SO_3_)_2_ | ~33 kPa | - | Energy Storage Mater. 2022, 44,  517 |
| PAM | SN | Zn(CF_3_SO_3_)_2_  DMSO | 48.8 kPa | - | Adv. Funct. Mater. 2022, 32, 2112540 |
| P(AM-*co*-SBMA) | SN | Zn(TOf)_2_ | 27.9 kPa | 50 kPa at 60% strain | Adv. Energy Mater. 2022, 12, 2202219 |
| ZSC-gel | *s*-IPN | ZnSO_4_  MnSO_4_ | Tensile modulus of 24.46 kPa | - | Adv. Energy Mater. 2020, 10, 2000035 |
| PAM/DMSO/CNF | *s*-IPN | ZnSO_4_  MnSO_4_ | 55.4 kPa | - | Chem. Eng. J. 2023, 464, 142607 |
| P(PEGMEA-AM)/PAM | *s*-IPN | ZnSO_4_ | 75 kPa | - | Chem. Eng. J. 2023, 463, 142535 |
| PAAm/agar/ Zn(CF_3_SO_3_)_2_ | DN | Zn(CF_3_SO_3_)_2_ | 78.9 kPa | 118.0 kPa at 60.0% strain | ACS Appl. Mater. Interfaces 2022, 14, 23452 |
| PAMPS/PAM | DN | ZnSO_4_ | 103 kPa | - | Chem. Eng. J. 2022, 446, 137021 |
| PAM/CMC/xanthan gum | DN | Zn(CF_3_SO_3_)_2_ | 34.9 kPa | 360.8 kPa 80% | J. Energy Chem. 2023, 86, 373 |
| Zn^2+^-CS/PAAM | DN | Zn(CF_3_SO_3_)_2_ | 80 kPa | - | Chem. Eng. J. 2023, 452, 139605 |
| CS/PASP/ZnSO_4_ | DN | ZnSO_4_ | 53.2 kPa | - | Nat. Commun. 2023, 14, 4435 |
| PAM/LA/PSBMA | Triple network (mentioned in article) | Zn(CF_3_SO_3_)_2_ | ~70 kPa | - | ACS Nano 2024, 18, 12355-12366 |
| **This work**  **PBCM-HE** | **Single network** | **ZnSO_4_**  **MnSO_4_** | **105 kPa** | **0.6 MPa at 80% strain** | **-** |


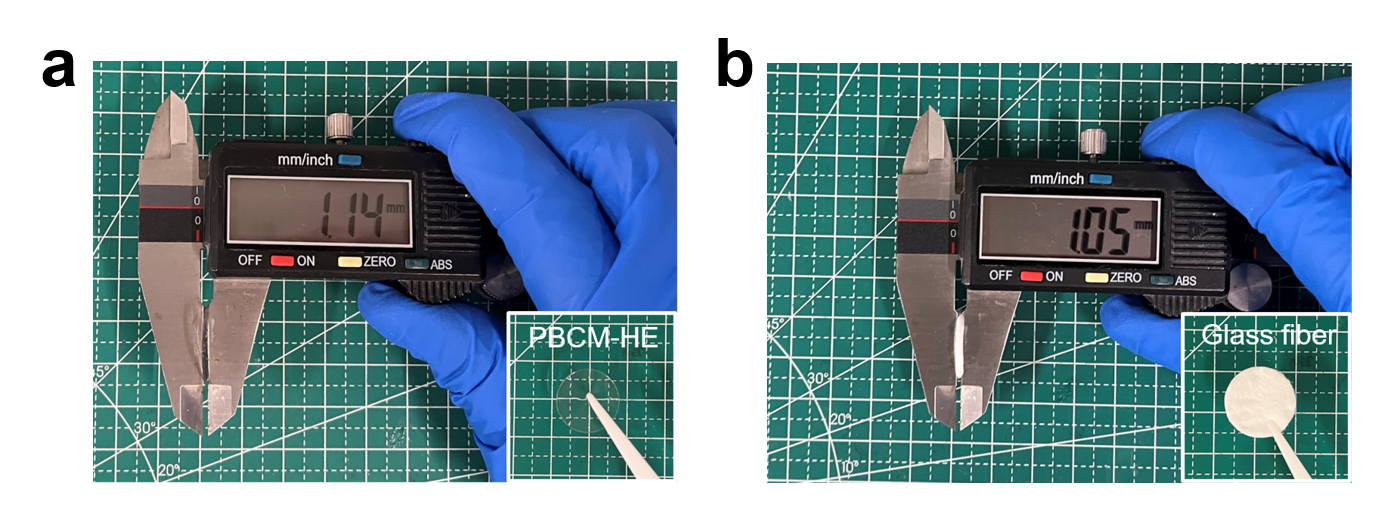


**Figure S7.** The thickness measurements of (a) PBCM*-*HE and (b) commercial glass fiber. (Digital images are shown in the insets)


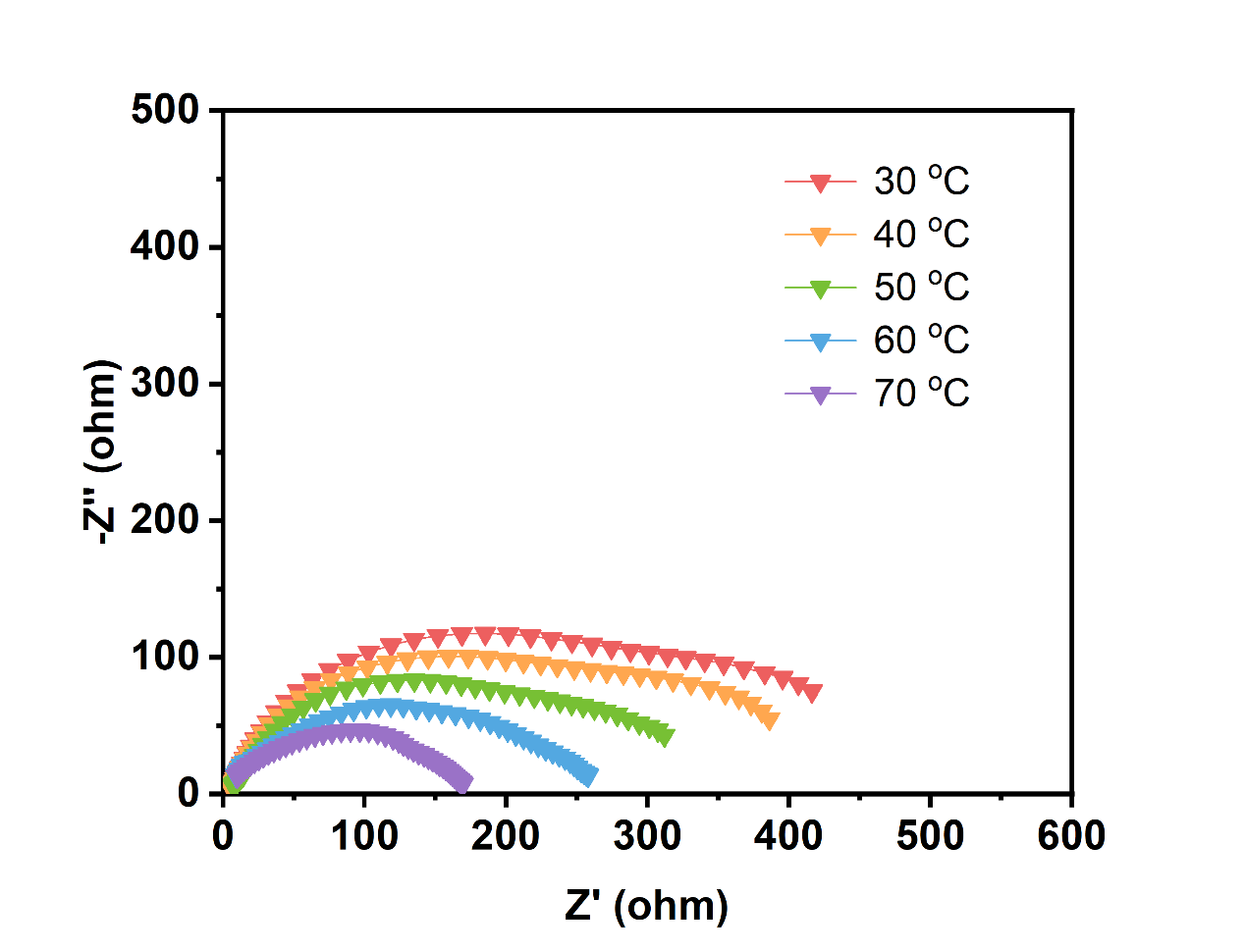


**Figure S8.** EIS curves of symmetric cells with PAM-HE at different temperatures.


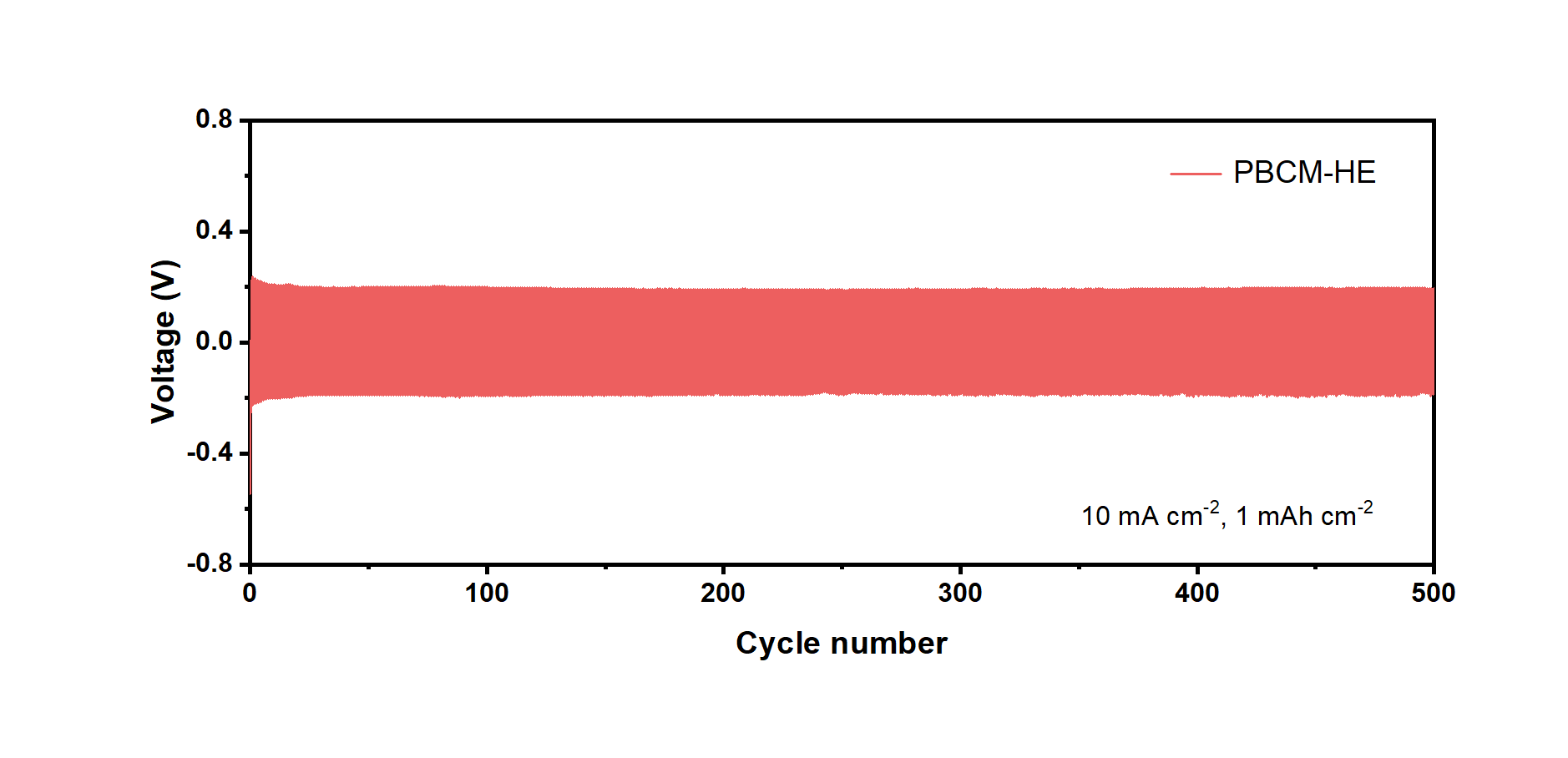


**Figure S9.** Zn plating/stripping curve of Zn||Zn symmetric cells in PBCM-HE under the current density of 10 mA cm^-2^.


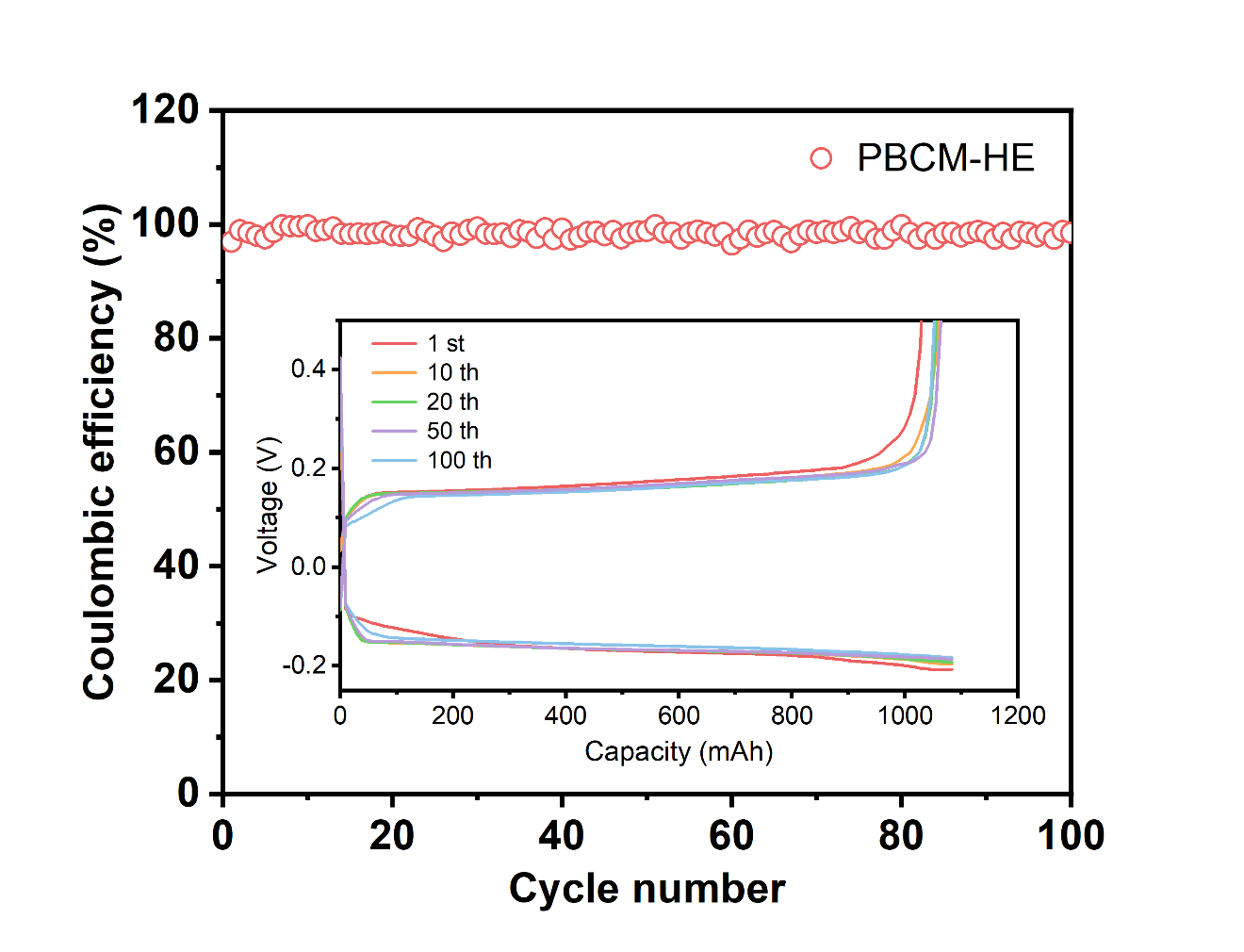


**Figure S10.** Coulombic efficiency of Zn deposition based on Zn||Cu asymmetric cells with PBCM-HE at the areal capacity of 1 mAh cm^-2^.


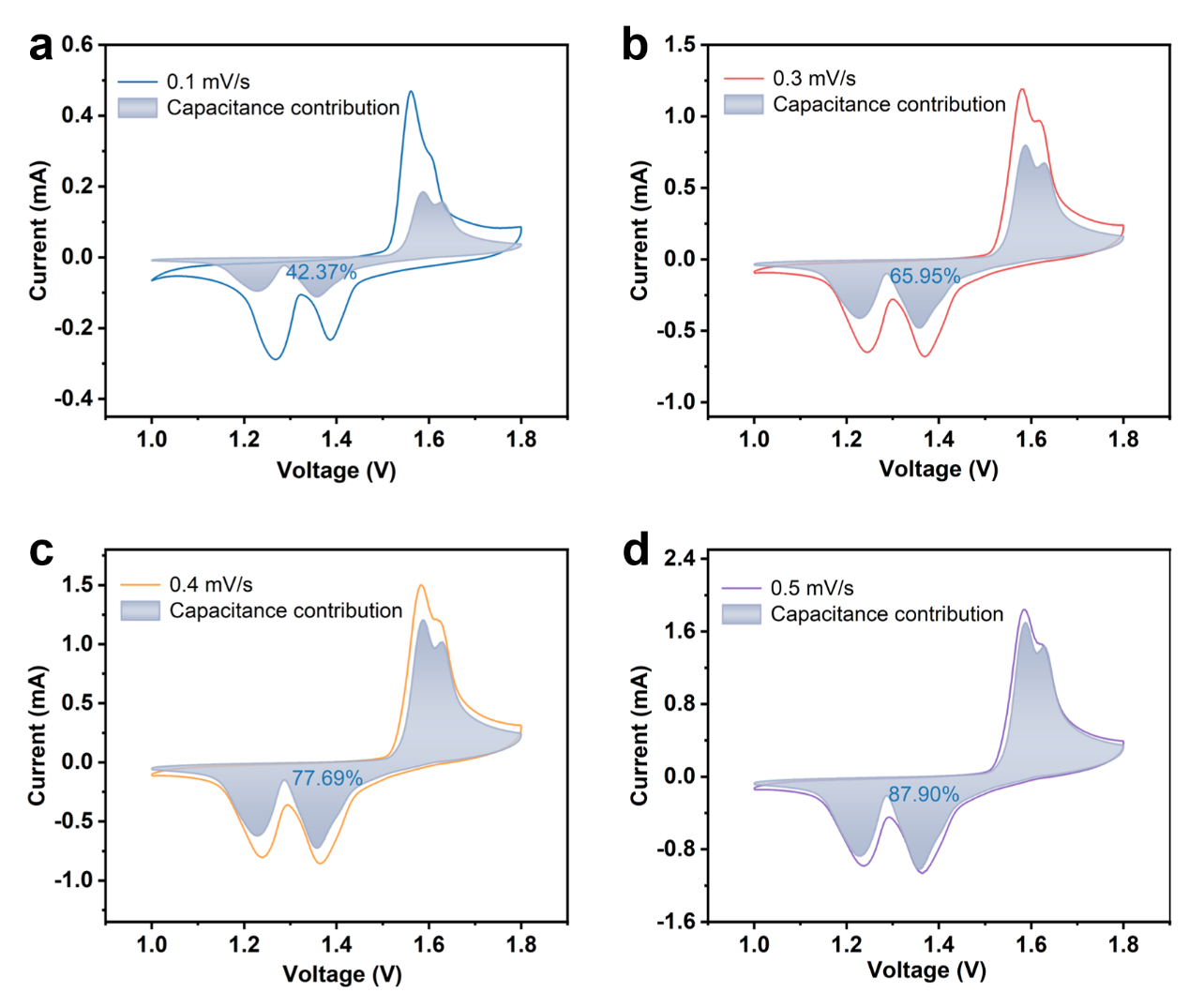


**Figure S11.** Capacitance contribution (blue part) and diffusion contribution (blank part) at (a) 0.1 mV s^−1^, (b) 0.3 mV s^−1^, (c) 0.3 mV s^−1^, (d) 0.5 mV s^−1^.


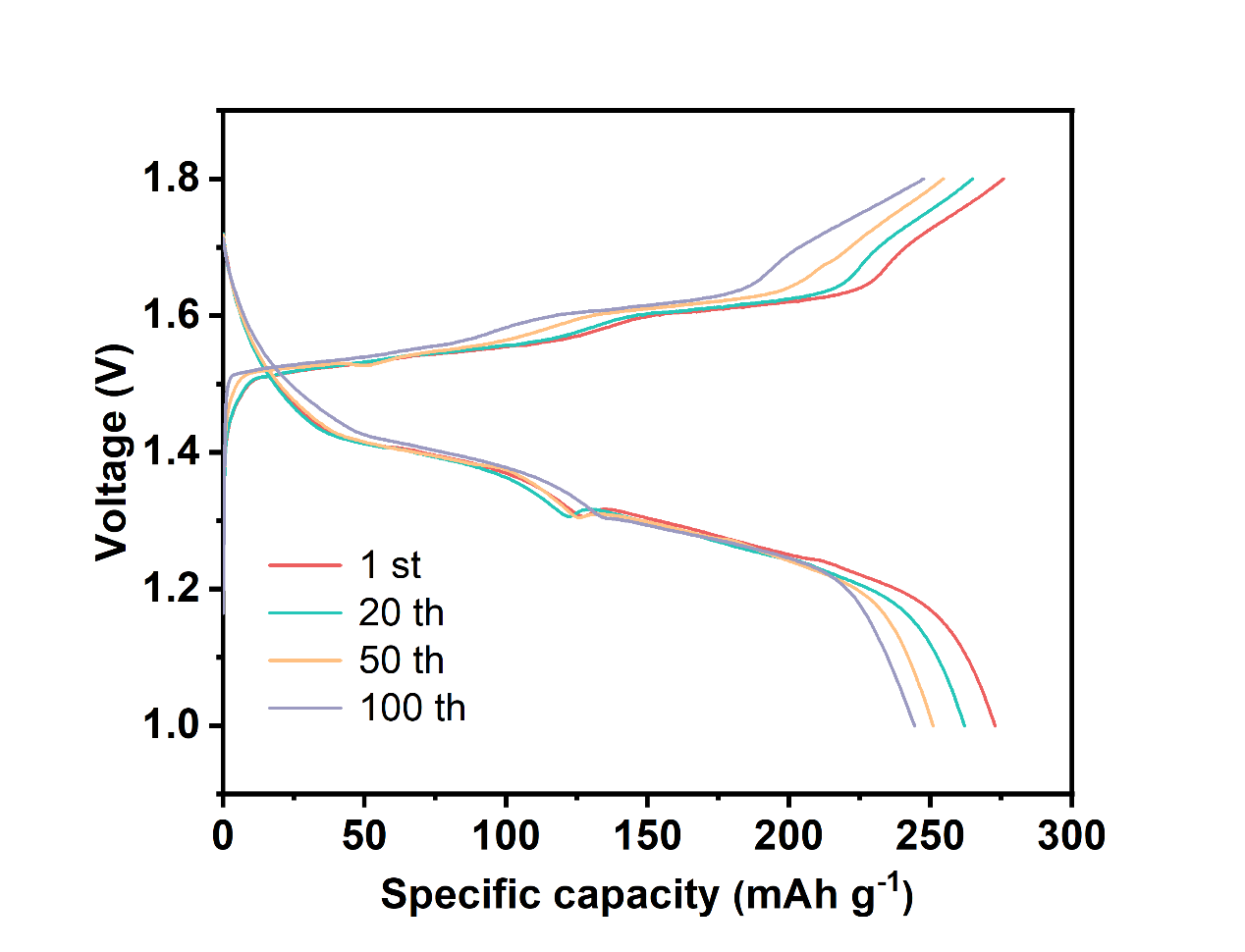


**Figure S12.** The GCD curves corresponding to different cycle numbers at 0.2 C.

**Table S2.** Comparison of cycle performance in Zn-MnO_2_ between PBCM-HE work and other reported related works. (ISC and RSC stand for initial specific capacity and residual specific capacity, respectively.)

| **Electrolyte type** | **Current density**  **(1 C=308 mA/g)** | **Cycle number** | **ISC**  **(mAh/g)** | **RSC**  **(mAh/g)** | **Reference** |
| --- | --- | --- | --- | --- | --- |
| Xanthan gum/ZnSO_4_/MnSO_4_ | 5.0 C | 1000 | - | 127 | J. Mater. Chem. A 2018, 6, 12237 |
| NFC/PAM hydrogel | 4.0 C | 1000 | ~200 | ~176.6 | Small 2018, 14, 1803978 |
| Zwitterionic sulfobetaine/cellulose gel (ZSC-gel) | 6.5 C | 1200 | 148 | ~133.8 | Adv. Energy Mater. 2020, 10, 2000035 |
| P(AM-DMAPS)/ Zn(ClO_4_)_2_ | 0.6 A/g | 1000 | - | 150 | Nano-Micro Lett. 2022, 14, 93 |
| PVDF-HFP gel | 1.0 C | 1000 | - | 124.6 | Adv. Funct. Mater. 2022, 2209463 |
| PVIPS/BC zwitterionic gel | 1.0 C | - | 230 | - | Adv. Sci. 2022, 9, 2104832 |
|  | 5.0 C | 1000 | - | 150 |  |
| PAMPS/PAM | 0.3 A/g | 500 | 150 | 60 | J. Mater. Chem. A 2022, 10, 3122 |
| PAAm/HNTs hydrogel | 0.2 A/g | 200 | ~340 | ~270 | Colloid Surface A 2022, 647, 129195 |
| TCOF-S-Gel | 1.0 C | - | 248 | - | Angew. Chem. Int. Ed. 2023, 62, e202312020 |
| Zn^2+^-CS/PAAM hydrogel | 3.0 A/g | 2000 | 172 | ~162.9 | Chem. Eng. J. 2023, 452, 139605 |
| Aniso-CMC hydrogel | 0.1 A/g | - | 288.9 | - | Proc. Natl. Acad. Sci. U.S.A. 2024, 121, e2322944121 |
| SA/EIDC gel electrolyte | 0.1 A/g | - | 247 | - | Energy Storage Mater. 2025, 74, 103903 |
| **This work**  **PBCM-HE** | **0.2 C** | **100** | **272.5** | **238.7** | **-** |
|  | **5.0 C** | **700** | **151.2** | **135.1** |  |

**References**

[1] H. Pan, Y. Shao, P. Yan, Y. Cheng, K. S. Han, Z. Nie, C. Wang, J. Yang, X. Li, P. Bhattacharya, K. T. Mueller, J. Liu, *Nat. Energy* **2016**, *1*, 16039.

[2] M. J. Abraham, T. Murtola, R. Schulz, S. Páll, J. C. Smith, B. Hess, E. Lindahl, *SoftwareX* **2015**, *1-2*, 19-25.

[3] J. Wang, R. M. Wolf, J. W. Caldwell, P. A. Kollman, D. A. Case, *J. Comput. Chem.* **2004**, *25*, 1157-1174.

[4] S. Grimme, C. Bannwarth, P. Shushkov, *J. Chem. Theory Comput.* **2017**, *13*, 1989-2009.

[5] T. Lu, F. Chen, *J. Comput. Chem.* **2012**, *33*, 580-592.

[6] Z. Liu, T. Lu, Q. Chen, *Carbon* **2021**, *171*, 514-523.
